# Synoptic Variation Drives Genetic Diversity and Transmission Mode of Airborne DNA Viruses in Urban Space

**DOI:** 10.1002/advs.202404512

**Published:** 2024-10-22

**Authors:** Aihua Deng, Junyue Wang, Lai Li, Ruilin Shi, Xuemin Li, Tingyi Wen

**Affiliations:** ^1^ State Key Laboratory of Animal Nutrition and Feeding, Ministry of Agriculture and Rural Affairs Feed Industry Centre China Agricultural University Beijing 100193 P. R. China; ^2^ State Key Laboratory of Microbial Resources Institute of Microbiology Chinese Academy of Sciences Beijing 100101 P. R. China; ^3^ Beijing Bio‐Feed Additives Key Laboratory Beijing 100193 P. R. China; ^4^ Department of Ophthalmology Peking University Third Hospital Beijing 100191 P. R. China; ^5^ Savaid Medical School University of Chinese Academy of Sciences Beijing 100049 P. R. China

**Keywords:** airborne viromes, genetic diversity, metabolic function, synoptic variation, transmission mode

## Abstract

Airborne viruses are ubiquitous and play critical roles in maintaining ecosystem balance, however, they remain unexplored. Here, it is aimed to demonstrate that highly diverse airborne viromes carry out specific metabolic functions and use different transmission modes under different air quality conditions. A total of 263.5‐Gb data are collected from 13 air samples for viral metagenomic analysis. After assembly and curation, a total of 12 484 viral contigs (1.5–184.2 kb) are assigned to 221 genus‐level clades belonging to 47 families, 19 orders, and 15 classes. The composition of viral communities is influenced by weather conditions, with the main biomarker being *Caudoviricetes*. The most dominant viruses in these air samples belong to the dsDNA *Caudoviricetes* (54.0%) and ssDNA *Repensiviricetes* (31.2%) classes. Twelve novel candidate viruses are identified at the order/family/genus levels by alignment of complete genomes and core genes. Notably, *Caudoviricetes* are highly prevalent in cloudy and smoggy air, whereas *Repensiviricetes* are highly dominant in sunny and rainy air. Diverse auxiliary metabolic genes of airborne viruses are mainly involved in deoxynucleotide synthesis, implying their unique roles in atmosphere ecosystem. These findings deepen the understanding of the meteorological impacts on viral composition, transmission mode, and ecological roles in the air that we breathe.

## Graphical abstract

Airborne viral communities are influenced by weather conditions with highly prevalent *Caudoviricetes* in cloudy/smoggy air and *Repensiviricetes* in sunny/rainy air, revealing different transmission modes under various air quality conditions. They carry diverse auxiliary metabolic genes mainly for the synthesis of nucleotide sugars, dNDP/dNTP, and cofactors, which could play crucial roles in viral DNA replication and survival in the extreme atmosphere.

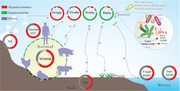

## 1 Introduction

Viruses originating from various environments are ubiquitous in air^[^ ^1^, ^2^ ^]^ and can spread through atmospheric aerosols, resulting in large‐scale biological migration and air pollution.^[^ ^3^, ^4^, ^5^, ^6^ ^]^ Thus, viruses in air have a detrimental impact on the environment and human health. Some viruses are known to cause diseases, such as influenza, severe acute respiratory syndrome (SARS), and coronavirus disease‐2019 (COVID‐19).^[^ ^6^, ^7^, ^8^, ^9^ ^]^ Despite the extreme conditions of strong radiation, nutrient scarcity, and variable temperatures of the atmosphere, it allows viruses to disperse over longer distances.^[^ ^10^, ^11^ ^]^ A large number of viable microorganisms have been reported to exist in stratospheric aerosol at altitudes greater than 40 km,^[^ ^12^ ^]^ indicating that aerosol transmission of viruses could occur through the atmosphere, besides person‐to‐person spread in local areas.^[^ ^13^ ^]^ Therefore, taxonomic and metagenomic research on airborne viruses is vital for understanding their roles in ecosystems and human health.

Research on airborne microorganisms has long relied on traditional culture methods.^[^ ^6^ ^]^ With the advancement of next‐generation sequencing (NGS), an increasing amount of microbial data has recently been obtained from a wide range of environments. To date, over 402709 metagenome‐assembled genomes have been reconstructed from diverse habitats, representing 85205 novel candidate species‐level operational taxonomic units spanning 181 phyla (https://gtdb.ecogenomic.org/stats/r214). This catalog expands the known phylogenetic diversity of microorganisms by over 44%.^[^ ^14^ ^]^ Additionally, the abundance of airborne bacteria and fungi varies seasonally,^[^ ^15^, ^16^ ^]^ and they change significantly, even within a short period of time, owing to local and regional meteorological conditions.^[^ ^17^, ^18^ ^]^ Moreover, areas with high traffic density, sewage pollution, and dust storms have significantly higher concentrations of airborne bacteria and fungi.^[^ ^19^, ^20^, ^21^ ^]^ These studies suggest that external environmental factors affect the abundance and dynamics of airborne microbes.

Compared to bacteria and archaea, our knowledge of viruses in nature, especially in the air, remains limited. For example, based on the public IMG/M (Integrated Microbial Genomes & Microbiomes) database, Nayfach et al. recently analyzed 15913 metagenomes from the oceans and other aquatic environments, and 2652 metagenomes from soils and other terrestrial environments. However, only 21 metagenomes have been obtained from atmospheric environments, indicating the difficulty in collecting airborne samples because of their extremely low density.^[^ ^14^ ^]^ Moreover, the lack of standard marker genes and detection tools limits our understanding of viruses in air. Cao et al. identified 0.1% viral reads in the microbial composition of smog air; however, only three viral species were identified: *Pseudomonas* phage F116, Adenovirus C and *Enterobacteria* phage P1.^[^ ^22^ ^]^ The seasonal composition of bacteria, fungi, and viruses from airborne environments in urban spaces has been reported, but only one *Pseudomonas* phage has been identified among 60 putative viruses.^[^ ^23^ ^]^ Prussin et al.^[^ ^24^ ^]^ and Whon et al.^[^ ^25^ ^]^ reported that the season change could significantly affect airborne viral abundance and community composition in some locations such as classrooms, offices, daycare centers, and some land districts. An inverse relationship between viral abundance and environmental factors (temperature and humidity) has been proposed, and seasonal cycles of temperature and absolute humidity might be the key factors controlling seasonal changes in viral abundance.^[^ ^25^ ^]^ Therefore, the community composition and abundance of airborne viruses are affected by complex environmental factors. Previous studies have focused on the seasonality of airborne viruses in some regions.^[^ ^25^, ^26^ ^]^ However, to our knowledge, the effects of different weather conditions on the abundance and community composition of airborne viromes have not yet been explored. Therefore, our current knowledge of the genetic diversity, transmission modes, and ecological roles of atmospheric viral communities remains limited.

In the present study, we conducted a metagenomic survey of air viromes under different weather conditions (sunny, cloudy, rainy, and smoggy days) in the near‐surface atmosphere of Beijing, China. Smoggy samples consisted of lightly polluted (LP) and moderately polluted (MP) groups according to the air quality index (AQI). A total of 263.5 Gb of metagenomic data were acquired, which revealed the relationship of the DNA viral community composition with meteorological changes and the underlying biomarkers of synoptic differences. A group of novel viruses was characterized by alignment of complete genomes and core genes. Furthermore, auxiliary metabolic genes (AMGs) and virus‐host linkages were analyzed to reveal the ecological roles and transmission mode of the atmospheric viral community, to the best of our knowledge, for the first time. This study improves our understanding of meteorological influences on viral composition and airborne transmission, with potential implications for public health.

## 2 Results

### 2.1 Overview of the Airborne DNA Viral Community

**Figure 1:**
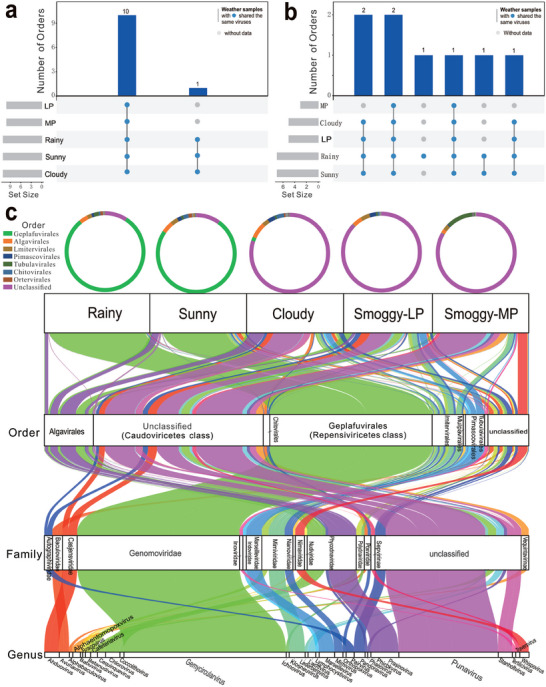
The composition of DNA viral communities in different kinds of weather. a) UpSet plot of the shared and specific dsDNA viruses. b) UpSet plot of the shared and specific ssDNA viruses. Set size refers to the original order numbers (n ≥ 3) of each weather. Blue points indicate data available for that weather. Gray points indicate that data is unavailable for that weather. Black lines indicate that the weather samples with blue points contained the same viruses. The numbers on the histogram show the shared or specific virus amounts. c) The genetic diversities of airborne viruses at the order/family/genus levels. The Sankey diagram shows the distribution of the top 30 most abundant DNA viruses at the genus level. The dominant viruses of cloudy and smoggy samples belong to the *Caudoviricetes* class with unclassified order and family. LP and MP indicate light and moderate air pollution, respectively.

A total of 699 433 931 clean reads were obtained from the viral DNA of sunny, cloudy, rainy, LP and MP samples (Table S1 and Figure S1, Supporting Information). Among them, 48 610 273 virus reads were identified with ratios of 2.86%–20.74% (Table S2, Supporting Information), which were then assembled and curated to obtain 16 021 599 viral contigs. After assessment and filtering by combining CheckV and Visorter2, 141 567 confident viral contigs were identified (Table S3, Supporting Information). By classification and curation, a total of 12 484 viral contigs (1.5–184.2 kb) from 13 airborne viromes assigned to 221 genus‐level clades belonging to 47 families, 19 orders, and 15 classes. Most viruses that dominated in these air samples belonged to the dsDNA (double‐stranded DNA) *Caudoviricetes* (54.0%) and ssDNA (single‐stranded DNA) *Repensiviricetes* (31.2%) classes. A total of 19 viral orders were identified from the 13 viromes, including eleven orders belonging to dsDNA viruses and eight orders belonging to ssDNA viruses. Under the five weather conditions, ten dsDNA and three ssDNA viral orders were observed (**Figure** **1**a,b; Table S4, Supporting Information).

Six abundant viruses were identified in five types of weather, and their relative abundances varied with weather conditions. Most viruses were assigned to *Geplafuvirales*, *Algavirales*, *Lmitervirales*, *Pimascovirales*, *Tubulavirales*, and *Chitovirales* at the order level. Notably, a group of viruses with unclassified order and family was assigned to highly diverse‐tailed phages (caudoviruses) that belong to the *Caudoviricetes* class (Figure 1c). Caudoviruses have been reported to be the most abundant virus in the gut viromes of humans and other animals.^[^ ^27^, ^28^, ^29^ ^]^ Surprisingly, the relative abundance proportions of *Caudoviricetes* were highly distributed on cloudy, LP and MP days (69.7–78.4%), but only accounted for 9.0–9.2% in rainy and sunny samples. The relative abundance of *Caudoviricetes* in smoggy (LP and MP) samples was significantly higher than that in rainy and sunny samples (*p* = 0.03, ANOVA).

The second most abundant virus was *Geplafuvirales* belonging to *Repensiviricetes class*, which was highly dominant in sunny and rainy samples (69.1–77.6%). However, these viruses were very low (0.1–1.4%) in the smoggy (LP and MP) and cloudy samples. The vast majority of genomoviruses have been identified from diverse environmental‐, animal‐ and plant‐associated habitats.^[^ ^30^, ^31^ ^]^ Other viral orders, including *Algavirales*, *Lmitervirales*, *Pimascovirales*, *Tubulavirales*, and *Chitovirales*, accounted for 1.4–4.0% in all air samples. As shown in Figure 1c, dsDNA caudoviruses were dominant in cloudy and smoggy (LP and MP) samples, whereas ssDNA genomoviruses were predominant in sunny and rainy samples.

### 2.2 Viral Community Structures were Shaped by Weather

**Figure 2:**
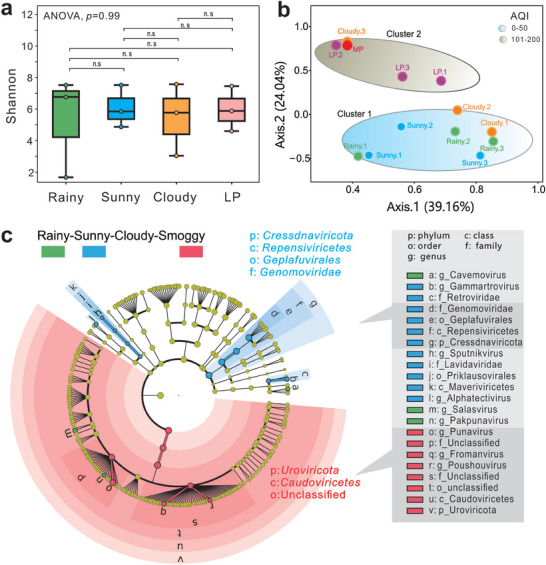
Viral diversity changes with weather. a) Shannon diversity index of sunny, cloudy, rainy, and LP samples. Horizontal lines within boxes represent medians. Tops and bottoms of boxes represent the 75th and 25th percentiles, respectively. Upper and lower whiskers extend 1.5 × past the interquartile range. There is no statistical difference in Shannon indices (n ≥ 3, *p* > 0.05). b) Contrasts in community composition based on Bray–Curtis distance. The distance between samples represents the dissimilarity of viral community composition, which is significant clustered by AQI values (n ≥ 3, *p* = 0.04). AQI record of all samples are shown in Table S1 and Figure S2 (Supporting Information). AQI values of 0–50 indicate excellent air quality. AQI values of 101–200 indicate light and moderate air pollution (Environmental Air Quality Standards GB3095‐2012). c) LEfSe (Linear discriminant analysis Effect Size) results on viral community evolutionary branch. The evolutionary branch diagram of different viruses, with circles radiating from inside to outside, represents the classification level from phylum to genus. Each small circle at different classification levels represents a classification at that level. The diameter of the small circle is proportional to the relative abundance. Species with no significant differences are uniformly colored yellow (n ≥ 3, *p* > 0.05). Red nodes represent viral groups that play an important role in the red group (Smoggy days, n = 4, *p* < 0.04). Blue nodes represent viral groups that play an important role in the blue group (Sunny days, n = 3, *p* < 0.04).

To investigate whether the number of viral species (richness) and their composition (evenness) in the air varied with the weather, we calculated the Shannon index. No significant differences in the Shannon indices (*p* = 0.99) suggested that the weather did not have a significant effect on the abundance and composition of the viral species (**Figure** **2**a). On the contrary, the principal coordinates analysis (PCoA) plots showed that the Bray‐Curtis distance of smoggy (LP and MP) samples was distinct from that of sunny and rainy samples (Figure 2b). Furthermore, the viral community structures in the three samples were clustered by air quality, which is usually evaluated by an indicator AQI. The viral community structures of repeatedly cloudy samples were distinct, mainly due to variable air quality on cloudy days. The AQI value of Cloudy‐3 was significantly higher than those of the other two samples in the cloudy group (*p* < 0.05), suggesting that the air quality of Cloudy‐3 was closer to light air pollution and thus clustered with the smoggy (LP and MP) samples (Figure S2, Supporting Information). Other meteorological factors, such as temperature and humidity, showed no statistically significant effect on the cluster of viral communities. The viral community structures in 13 airborne samples were noticeably clustered by the air quality among various meteorological parameters (Figure 2b). The most abundant virus *Caudoviricetes* in smoggy samples might represent the viral community in poor air as shown in Figure 1c.

Based on the linear discriminant analysis effect size (LEfSe) analysis, synoptic changes were the main drivers of changes in viral community structures among all samples (Figure 2c). Among the significantly different viruses, *Caudoviricetes* from the phylum *Uroviricota* was identified as the most important biomarker, and its relative abundance was significantly increased on smoggy days with poor air quality parameters (Figure 1c). The second most abundant virus, *Repensiviricetes* from the phylum *Cressdnaviricota*, also contributed to viral community differences (Figure 2c). These results showed that the conditions of sunny, rainy, and smoggy weather could affect viral community structures but did not have a significant effect on the abundance and distribution of viral species. Thus, dsDNA *Caudoviricetes* was identified as the most important difference for synoptic changes among all samples, in agreement with a significant increase in their relative abundance on smoggy days with poor air quality parameters.

### 2.3 Genetic Diversity of Airborne *Caudoviricetes* and Identification of New Viruses

**Figure 3:**
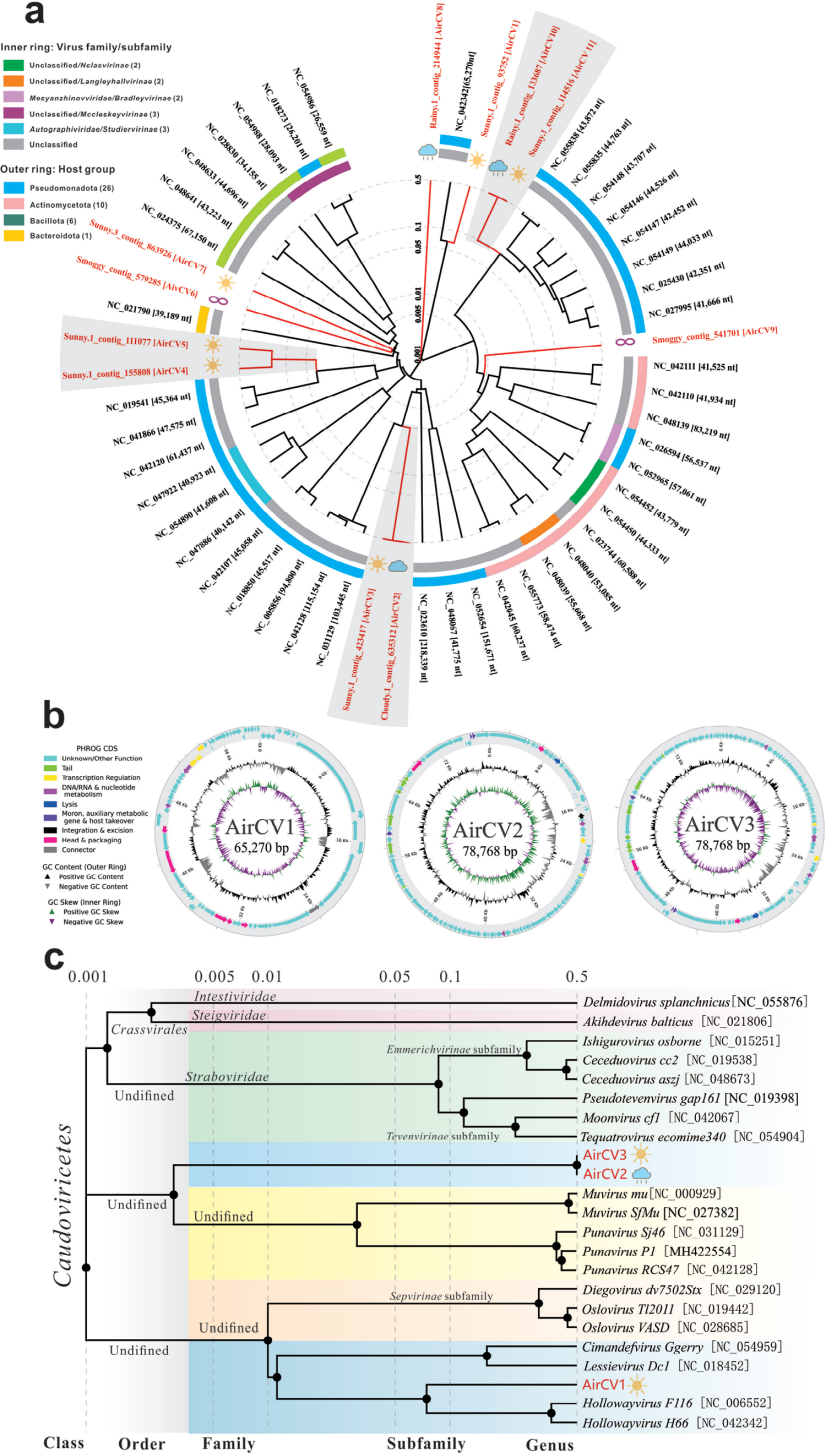
Phylogenetic tree and genomic divergence of caudoviruses. a) Phylogenetic tree of *Caudoviricetes*. Branch lengths are linearly scaled and represent genomic distance. Reference sequences are colored in black, whereas unique contigs from different weather samples are colored in red. b) Genomic organization of complete genomes of AirCVs 1–3. ORFs with functional prediction results are shown in different colors. c) Phylogenetic tree of new AirCVs 1–3 and their closest viruses of class *Caudoviricetes*. Branch lengths are log‐scaled and represent genomic distance. Sequence similarity among the representative caudoviruses and AirCVs are presented in the supplementary materials.

To assess the genetic diversity of airborne viruses, phylogenetic analyses were performed on viruses assigned to *Caudoviricetes* and *Geplafuvirales*, which predominantly accounted for 13 viromes. After screening *Caudoviricetes* contigs with 95% and higher completeness, sequences larger than 10 kb and unclassified order/family/genus levels, a total 11 high‐quality contigs with 43–81 kb sequences were selected as unique airborne viruses to construct a proteomic tree for *Caudoviricetes* based on genome‐wide identities (**Figure** **3**a). All viral contigs were distant from the known reference sequences, forming separate clades from the representative reference sequences. These clades were distant from each other and included sequences from samples of all the weather conditions.

To assess the genomic diversity of AirCVs (Air‐associated caudoviruses) of five weathers, amino acid sequence alignments were compared to identify the divergence between airborne viral contigs and other most similar viruses in the phylogenetic tree (Figure 3; Figures S3–S7, Supporting Information). Phylogenetic analysis indicated that AirCV1 comprised a separate clade in the *Podoviridae* group clustering with *Hollowayvirus* with 50% identity to the whole sequence, demonstrating their distant evolutionary relationship (Figure 3; Figure S3, Supporting Information). AirCV2 clustered with AirCV3, sharing >95% sequence identity; however, their alignment identities with *Muvirus* and *Punavirus* were lower than 40% (Figure 3; Figure S4, Supporting Information). AirCV4 clustered with AirCV5, forming a clade close to the *Acinetobacter* phage, with 4–7% coverage for similar sequences (Figure 3; Figure S5, Supporting Information). Genomic BLAST showed that AirCVs 6–9 had no homologous viruses and their similarities with each other were extremely low (Figure 3; Figure S6, Supporting Information). Although AirCV10 clustered with AirCV11, sharing >95% identity and therefore belonging to the same taxonomy, they had low alignment with *Xanthomonas* phage because nearly half of the contigs were unmatched (Figure 3; Figure S7, Supporting Information). Eleven airborne *Caudoviricetes* were in general phylogenetically distant from the known reference sequences belonging to different clusters, highlighting the important genomic diversity of *Caudoviricetes* in air.

Based on a BLASTn search, three airborne *Caudoviricetes* viruses (AirCV1, AirCV2, and AirCV3) with complete genomes showed 40–50% similarity to known viruses. Their genome organization is shown in Figure 3b. Bioinformatics analysis indicated that AirCV1 contained a dsDNA genome of 65 270 bp with a G+C content of 50% and 57 open reading frames (ORFs). AirCV2 and AirCV3 both contained a dsDNA genome of 78 768 bp with a G+C content of 45%. Genomic annotation of AirCV2 and AirCV3 predicted 110 and 109 ORFs, respectively. Most of these ORFs showed low identity (<25%) with known proteins and were predicted to encode hypothetical proteins with unknown functions (Figure 3b). The putative functions of some proteins include head and packing, transcription regulation, DNA/RNA nucleotide metabolism, lysis, and AMGs. These proteins exhibited 25–77% identity with conserved domains and structures in the UniProtKB/Swiss‐Prot database (Table S5, Supporting Information). Known AirCV2 and AirCV3 proteins perform the same functions, except for a specific N‐6 DNA methylase (N6_Mtase, Figure S8, Supporting Information). These viral proteins may play important roles in molecular function and cell composition.

Although many characteristics are important in determining taxonomic relationships, pairwise sequence alignment similarity and phylogenetic relationships have become the primary characteristics for defining and distinguishing viral taxa. Owing to the lack of a single conserved viral marker gene, such as 16S rRNA in bacteria, a single conserved gene cannot be aligned to any homologous hit. It is not sufficient to completely classify it as a new virus, because known viruses have a large number of unknown functional genes. Thus, new viruses are usually aligned at the genome‐wide level. A phylogenetic tree of AirCVs 1–3 and the representative viruses of their closest genera was constructed based on the full‐genome sequences (Figure 3c). AirCV1 was the most similar to *Hollowayvirus* genera, forming a potential new clade belonging to an undefined order in the *Caudoviricetes* class and comprising a clade separate from other related viruses of the *Sepvirinae* subfamily (Figure 3c). Notably, AirCV2 and AirCV3 comprised a clade separate from other related viruses in the *Caudoviricetes* class. As shown in Figure S4 (Supporting Information), the genomes of AirCV2 and AirCV3 were highly similar, with >95% overall DNA sequence identity. However, they separately shared only 33.4% to 45.8% identity with the *Punavirus* and *Muvirus* genera belonging to an undefined family. The results showed that AirCV2 and AirCV3 from the airborne samples were most similar to those of the *Punavirus* and *Muvirus* genera, possibly forming a novel candidate order.

### 2.4 Genetic Diversity of Airborne *Geplafuvirales* and Identification of New Viruses

**Figure 4:**
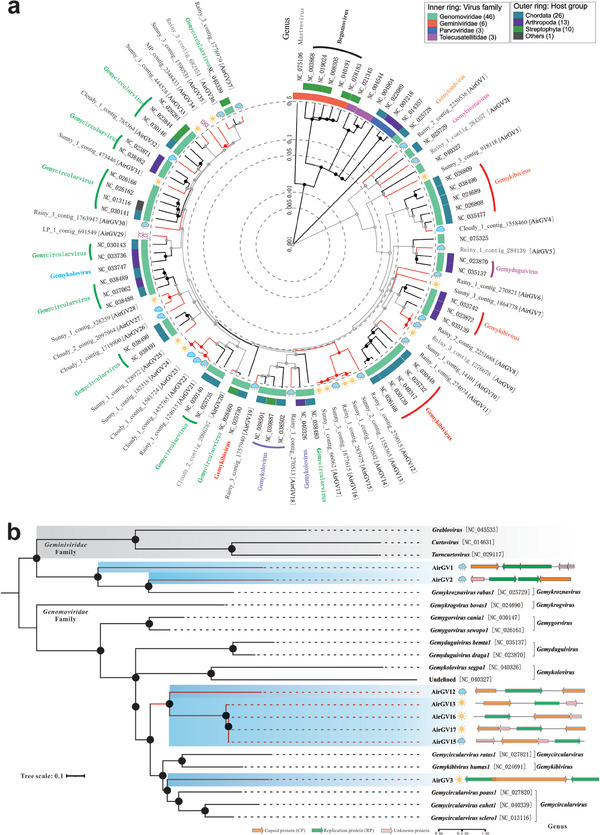
Phylogenetic tree and genomic divergence of genomoviruses. a) Phylogenetic tree of *Geplafuvirales*. Branch lengths are linearly scaled and represent genomic distance. Reference sequences are marked with black lines and indicated with genus names, whereas viral contigs from different weather samples are colored with red lines. b) Phylogenetic tree of new AirGVs with available genomoviruses based on the Rep proteins. The tree scale shows the number of amino acid substitutions per site. Genomic organizations of AirGVs are shown in the tree. Sequence similarity among the representative genomoviruses and AirGVs are presented in Figure S14 (Supporting Information).

*Geplafuvirales* has been divided into *Genomoviridae* and *Geminiviridae* families, including genomoviruses with small circular ssDNA genomes of 2.0–2.4 kb encoding a replication‐associated protein (Rep) and a capsid protein (CP).^[^ ^31^ ^]^ A phylogenetic tree was constructed for ssDNA *Geplafuvirales* viruses using 37 unique viral contigs from the air samples named as AirGVs (Air‐associated genomoviruses). As shown in **Figure** **4**a, high‐quality contigs and viral reference sequences were obtained from *Genomoviridae*. Some viral contigs from different weather conditions were distant from the known references. Most importantly, the sequences of AirGVs 8–10 and 13–14 from the sunny and rainy samples tended to cluster into a clade with a high level of identity (>95%). The identities of a large part of the sequences from cloudy and MP samples were only 20–70%. This indicates that the airborne viral genetic pool may be consistent between the sunny and rainy samples. As shown in Figures S9–S13 (Supporting Information), twenty‐five viruses clustered with *Gemykibivirius* (AirGVs 4 and 8–11)*, Gemyduguivirus* (AirGV5), *Gemykolovirus* (AirGVs 18–19) and *Gemycircularvirus* (AirGVs 20–37), sharing 50–100% identity with genome‐wide sequences. The alignment identities of the remaining viruses AirGVs 1–3 and 12–17 clustered with references were only 30–60%.

A phylogenetic tree of AirGVs 1–3, 12–17 and the representative viruses of their closest genus from *Genomoviridae* was established based on the alignment of the Rep protein and whole‐genome sequences (Figure 4b; Figure S14, Supporting Information). These viruses had typical characterization of genomoviruses with circular, ssDNA genomes of 2.0 to 2.4 kb, encoding Rep proteins, CP proteins, and some unknown proteins. The complete genomes of the 9 AirGVs showed 30–60% similarity with known viruses in GenBank, based on a BLASTn search (Figure S13, Supporting Information). To investigate the relationship with known genomoviruses, a phylogenetic tree based on the full genomic sequence and the Rep protein showed that AirGV1 and AirGV2 clustered with *Gemykroznavirus* (Figure 4b; Figure S14, Supporting Information). However, their distant relationships demonstrate that AirGV1 and AirGV2 could be novel candidate genomoviruses at the genus level. Furthermore, AirGV3 shared sequence similarity with an undefined virus by complete genomic alignment and formed a separate group at the genus level based on evolutionary trees of both the whole genomes and Rep proteins. Notably, AirGVs 12–17 from rainy and sunny days clustered into a new group at the genus level, for their 39–55% similarity with known viral genomes and 42–46% similarity to known Rep proteins. It has been reported that the members of a genomovirus in the same species should share >78% genome‐wide pairwise identity.^[^ ^32^ ^]^ Therefore, AirGVs from airborne samples were identified as novel candidate genera of the *Genomoviridae* family. The classified sequences were phylogenetically distant from known viruses in the databases, revealing abundant viral diversity in air.

### 2.5 Diverse Auxiliary Metabolic Functions of Airborne Viromes

**Figure 5:**
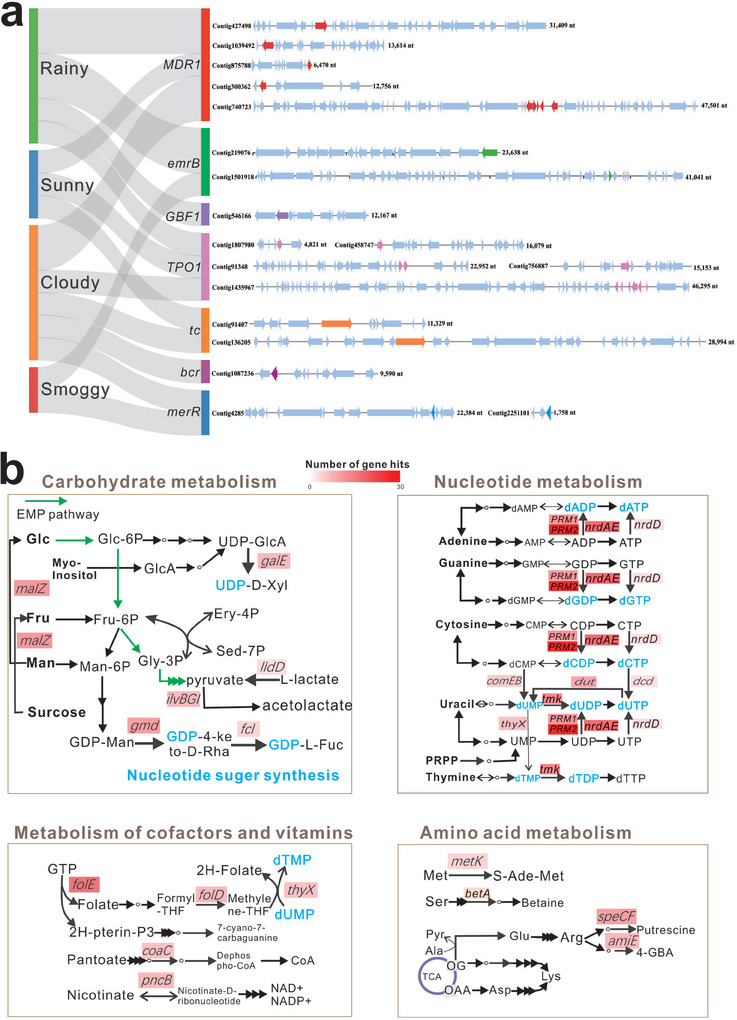
Predicted functional genes and related pathways of the airborne viromes. a) Antimicrobial resistance genes (ARGs) carried by airborne viruses in different weather conditions. The genomic organization of representative ARG‐carrying viral contigs. ARG is highlighted with the relevant color. b) Representative pathways associated with auxiliary metabolic genes (AMGs) of airborne viruses. Main pathways with gene count ≥3 were highlighted in different colors. The ORFs from confident contigs (1.5–184.2 kb) were functionally predicted by KEGG analysis. For further validation, ORFs were imported into HMMScan (e < 1e−5 and bit score >50) for annotation based on the Pfam database. Putative AMGs were manually checked and compared with reported AMGs.

High‐confidence ORFs from the viral genomes, based on KEGG analysis and HMMScan, are shown in Figure S15 (Supporting Information). The validated genes present in airborne DNA viromes are mainly involved in genetic information processing, signaling/cellular processes, metabolism, and functions related to infectious/immune diseases. Viruses proliferation is closely linked to genetic information processing (such as replication and repair, transcription and translation), signaling and cellular processes (such as signal transduction, folding, sorting, degradation, cell growth, and death) and diseases (such as infectious viral/bacterial disease, immune disease, and drug resistance). Among 12 848 viral contigs, 30 antibiotic resistance genes (ARG) were found in 18 contigs (**Figure** **5**a; Table S6, Supporting Information). Several major ARG classes, including MDR1, EmrB, GBF1, TPO1, and Tc are prevalent in airborne viromes. No significant differences were observed between the different weather conditions. Although airborne viromes carry only a few types of ARGs, their potential route of transmission is important for environmental safety.

Most metabolism‐related viral genes are categorized into metabolic pathways of carbohydrates, nucleotides, cofactors, vitamins, and amino acids. However, the specific auxiliary metabolic functions of the airborne viromes remain unclear. Figure 5b shows the metabolic pathways containing gene hits ≥3. Most genes involved in carbohydrate metabolism, such as *galE*, *gmd*, and *fcl*, were involved in the biosynthesis of nucleotide sugar. Additionally, the gene *malZ* encodes a protein involved in the conversion of various sugars/phosphorylated sugars, whereas *ildD* and *ilvBGI* encoding proteins are involved in pyruvate metabolism. Almost all genes predominantly participated in the conversion of phosphorylated ribonucleosides (NDP and NTP) to phosphorylated deoxyribonucleosides (dNDP and dNTP), which played a crucial role in viral DNA replication (Figure 5b). Genes of *folE*, *folD*, and *thyX* were involved in folate metabolism and dNTP synthesis, whereas genes *coaC* and *pncB* were involved in the synthesis of CoA, NAD^+^, and NADP^+^ cofactors in cofactor and vitamin metabolism. Genes involved in amino acid metabolism are mainly involved in the degradation of various amino acids, such as methionine, serine, and arginine. Among these putative AMGs, the *fcl*, *pncB*, *metK*, *nrdA*, and *dut* have been reported as common AMGs in previous studies.^[^ ^27^, ^33^ ^]^ Furthermore, the *nrdA* gene encoding ribonucleoside–diphosphate reductase for dNTP synthesis using NTP was an experimentally verified gene for viral replication.

These results demonstrated that airborne viruses could carry diverse AMGs that are responsible for the synthesis of nucleotide sugars, dNDP, dNTP, cofactors, and amino acid degradation/utilization. These specific metabolic functions supplied the necessary materials and energy for viral DNA replication and proliferation, implying the distinctive ecological role of airborne viruses in atmospheric habitats.

### 2.6 Virus‐Host Linkages and Transmission Mode of Airborne Viruses

**Figure 6:**
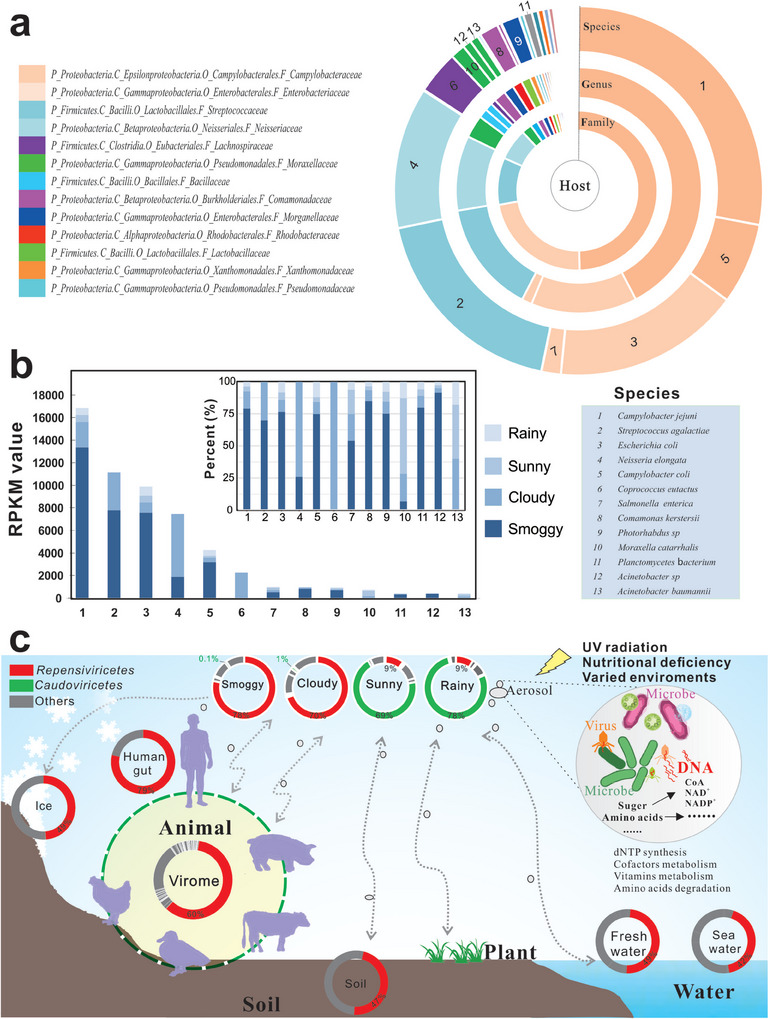
Virus‐host linkages and transmission mode of airborne viruses. a) The diversity and sources of the predicted hosts of airborne viruses at the family/genus/species levels. b) Species‐level host taxonomy and proportion of four weather conditions in the top 13 abundant bacterial hosts. c) The proposed mode of viral transmission between air and Earth's surface environments/living organisms.

It was interested to note that *Caudoviricetes* were widely present in the cloudy, LP and MP samples. To identify the sources of the viruses in air, we conducted further analyses of host diversity and origin. Among the possibly eukaryotic hosts, *Baikalvirus*, *Bracovirus*, *Sputnikvirus*, and *Ichnovirus* with 0.06% abundance that have been reported in parasitizing hosts,^[^ ^34^ ^]^ were putatively pathogenic to humans based on the viral protein families.^[^ ^35^ ^]^ *Caulimoviridae* with 1.94% abundance was putatively pathogenic to the plants *Manihot* and *Glycine* (Table S7, Supporting Information) (*36*). Surprisingly, 83.56% of the viral hosts belonged to *Campylobacteraceae*, *Enterobacteriaceae*, *Streptococcaceae*, and *Neisseriaceae* families, which are typically found in animal guts and other environments (**Figure** **6**a). The other 13.27% of the viral hosts belonged to families, such as *Fabaceae*, *Lachnospiraceae*, *Moraxellaceae*, *Bacillaceae*, *Comamonadaceae*, *Morganellaceae*, *Rhodobacteraceae*, *Lactobacillaceae* and *Xanthomonadaceae*. These microorganisms were found in various natural environments, such as the gut, soil, water, plants, and animals.

Among the top 13 most abundant bacterial hosts at the species level, *Campylobacter jejuni*, *Streptococcus agalactiae*, *Escherichia coli*, *Campylobacter coli*, *Comamonas kerstersii*, *Photorhabdus sp*., *Planctomycetes bacterium*, and *Acinetobacter sp*. were frequently identified in smoggy samples with >60% frequency (Figure 6b). *Campylobacter* and *Escherichia*, which are Gram‐negative bacteria often found in the guts of mammals and poultry had a high frequency of >70% in smoggy samples. Furthermore, *Salmonella enterica* often found in animal guts and other environments occurred at a frequency of 54% in smoggy samples. Besides, *Neisseria elongate* and *Moraxella catarrhalis* had a low frequency of 7–26% in smoggy samples, whereas *Coprococcus eutactus* only existed in cloudy samples. Thus, a high prevalence of airborne viruses was predicted to infect *Campylobacterales* and *Enterobacterales*, particularly on smoggy days.

The results indicated that microbial viruses were exchanged between the urban space and the Earth's surface environments/living organisms (Figure 6c). Viruses from plants, animals, water and soil are frequently transmitted into the Earth's atmosphere and could be exchanged in both bottom‐up and top‐down directions. Notably, *Repensiviricetes* and *Caudoviricetes* exhibit dynamic changes under different weather conditions. The abundances of *Repensiviricetes* are relatively high in rainy and sunny air samples (69.1–77.6%) but extremely low in cloudy and smoggy samples (0.1–1.4%). Contrary to the above results, low abundances of *Caudoviricetes* (9.0–9.2%) were observed in rainy and sunny samples, whereas cloudy and smoggy samples contained 69.7% and 78.4% *Caudoviricetes*, respectively (Figures 1, 6). According to previous reports, the content of *Caudoviricetes* is less than 50% in soil, freshwater, seawater, and ice.^[^ ^37^, ^38^ ^]^ However, a high dominance of *Caudoviricetes* (60–79%) has been reported in viromes of the human gut and animals,^[^ ^37^, ^39^ ^]^ similar to the high prevalence of *Caudoviricetes* in urban air on cloudy and smoggy days. Furthermore, viral hosts often found in the guts of mammals and poultry also showed a high frequency > 50% in the smoggy samples. Thus, viral transmission through humans and animals may be a mode of transmission in urban areas on cloudy and smoggy days.

## 3 Discussion

Viruses are ubiquitous in the air and their distribution varies temporally and spatially.^[^ ^2^, ^25^ ^]^ Although microbial abundance and community composition in air are generally thought to be shaped by environmental factors, knowledge of airborne viruses in diverse habitats remains largely unknown. Viral ecology benchmarking has recommended that a contig length threshold is necessary to improve confidence. Previous studies on viromes have used contigs larger than 1.0, 5.0, or 10.0 kb.^[^ ^27^, ^28^, ^40^ ^]^ In this study, we compared the effects of different sequence sizes (≥0.3, 1.0, 1.5, 2.0, 5.0, and 10.0 kb) in merged viral communities (Figure S16, Supporting Information). The viral metagenome diversity decreased as contig length increased, while viral abundances greatly varied with different contig length threshold. It is worth noting that *Repensiviricetes* did not exist in these viral communities with sequences larger than 5.0 and 10.0 kb, which was caused by small circular ssDNA genomes of 2.0 to 2.4 kb for *Repensiviricetes* class.^[^ ^31^ ^]^ To our knowledge, small viral sequences with complete genomes also have important implications for understanding viral ecology and evolution. Considering the completeness, diversity and abundance of airborne viromes in different weather samples, the viral community at contig length thresholds of 1.5 kb was used. After data curation, we comprehensively investigated the diversity of airborne viruses, their ecological roles and their transmission modes under different weather conditions. Noticeable clustering of the DNA viral community structures was found in the sunny, rainy, and smoggy samples, demonstrating *Caudoviricetes* as the main biomarker of synoptic differences (Figures 1, 2). Previous studies reported that airborne viruses were affected by season, with a dominance of plant‐associated ssDNA *geminivirus*‐related viruses.^[^ ^25^ ^]^ In daycare centers, human‐associated viruses were dominant in winter, whereas plant‐associated viruses were dominant in summer.^[^ ^26^ ^]^ Unlike previous reports, the effect of different weather conditions on the abundance and community composition of airborne viromes was reported for the first time in this study, revealing the dominance of the dsDNA *Caudoviricetes* on cloudy and smoggy days (70–78%) and ssDNA *Repensiviricetes* on rainy and sunny days (69–78%).

Amplification methods have been reported to bias sequence composition toward ssDNA viruses.^[^ ^41^ ^]^ However, some other studies suggested no significant bias in ssDNA abundance after amplification. For example, it has been reported that a 30‐min short multiple displacement amplification (sMDA) was used to limit the biases toward ssDNA.^[^ ^28^ ^]^ Another study found no significant difference in viral recovery between non‐amplified and amplified gut viromes.^[^ ^42^ ^]^ Furthermore, a gut virome study without whole‐genome amplification showed 99.2% dsDNA and ssDNA prokaryotic viruses, which is in line with amplification studies.^[^ ^43^ ^]^ In the present study, airborne viromes could not be obtained without amplification, owing to the scarcity of air samples. However, a 15‐min amplification was used in this study, which, according to above mentioned studies, could not induce significant biases of ssDNA. Furthermore, a thorough comparison of viral abundances in this study supports the conclusion that amplification does not increase ssDNA abundance. As shown in Figures 1, 6, ssDNA *Repensiviricetes* existing in all samples were predominant in sunny and rainy samples (69–78%). In smoggy (LP and MP) and cloudy samples, ssDNA *Repensiviricetes* accounted for only 0.1–1.4%. Furthermore, ssDNA viruses in smoggy and cloudy weather account for only 2.3% of all classified contigs. Thus, the abundance of ssDNA viruses in the classified contigs differed in various weather samples. As a cross‐reference for different samples, the results further showed that amplification in this study did not reflect the enrichment of ssDNA viruses.

Compared to the current understanding of viromes in soil and marine environments, data on airborne viromes have remained relatively deficient due to technological difficulties.^[^ ^4^, ^14^ ^]^ The lack of standard marker genes for viral taxonomy resulted in a high proportion of unknown sequences (40–90%) in previous virome studies.^[^ ^44^, ^45^ ^]^ Although we successfully collected and concentrated airborne samples to obtain high‐quantity viral contigs using the bioaerosols and dusts sampling (BANDS) payload,^[^ ^46^ ^]^ ≈25% of the sequences in the 13 viromes remained unclassified. Considering the easy degradation of RNA, this study mainly focused on airborne DNA viruses because of 48‐h collection time. Therefore, the study of airborne RNA viruses may be improved by optimization of collection techniques in future research. The high‐quality sequences assigned to *Caudoviricetes* and *Repensiviricetes* viral classes were distantly related to known references, suggesting a high diversity of viruses in the near‐surface atmospheres (Figures 1, 2, 3, 4). Previous airborne microbiomes have been found to contain caudoviruses or genomoviruses in different seasonal samples^[^ ^25^, ^26^ ^]^; however, the new viruses could not be characterized in detail owing to the lack of complete genomes. In this study, the complete genomes of the dominant viruses were obtained, and were identified as 12 new candidate viruses of the *Caudoviricetes* order at the order/family/genus level and of the *Gemykroznavirus* genus at the genus/species level (Figures 3, 4), revealing the diversity and main biomarker of airborne viromes under different weather conditions.

Viruses can evolve their diverse metabolic systems to facilitate infection and their survival in ecosystems^[^ ^27^, ^28^ ^]^; however, little is known about viruses in the atmospheric environment. To understand the potential ecological impact of viruses in air, we analyzed specific AMGs of airborne viromes (Figure 5). Auxiliary genes are classified into metabolic pathways of carbohydrates, nucleotides, cofactors, vitamins and amino acids, which are similar to those of viruses from marine and rumen biofilms.^[^ ^27^, ^33^ ^]^ The most prevalent metabolic genes in rumen viruses encode DNMT1, a DNA (cytosine‐5)‐methyltransferase and glycoside hydrolases involved in host defense and feed fiber digestion.^[^ ^27^ ^]^ The obvious difference is that airborne viruses have numerous genes related to the synthesis of nucleotide sugars, dNDP and dNTP, which play crucial roles in DNA replication (Figure 5). Furthermore, genes involved in cofactor synthesis and amino acid degradation and utilization were identified. Overall, airborne viruses have evolved efficient strategies to harness limited substrates and energy for viral DNA replication and proliferation, thus facilitating their adaptation to extreme atmospheric conditions, including nutritional deficiency, strong radiation, and varied temperatures.

Despite the diverse and distantly related genetic relationships of airborne viromes, *Geplafuvirales* in air under different weather conditions, shared a common genetic pool. Similar results have been reported for soil viromes,^[^ ^47^ ^]^ which showed that sequences of small ssDNA viruses from different mangrove soil samples formed clades separate from *Geminiviridae* (a family of *Geplafuvirales*). The viral genetic pool was consistent across the mangrove habitats because of the mixing and connectivity effects of marine tides. Although environmental conditions differ under distinct weather conditions, viruses may circle over a period of sampling in the air. We argue that such events likely resulted in a consistent phylogenetic relationship between *Geplafuvirales* in the air samples studied. For airborne viromes, the consistent genetic information of *Geplafuvirales* accounted for the transmitted role of air (Figure 4). Notably, highly diverse tailed‐phages (caudoviruses), which were previously reported to be highly abundant in the human gut and animal viromes,^[^ ^28^, ^37^ ^]^ were also highly abundant in cloudy and smoggy air (Figures 1, 6). The distribution of viral hosts was largely linked to *Campylobacterales* and *Enterobacterales*, further confirming viral transmission between air and living organisms and other environmental entities (Figure 6). Caudoviruses are more dominant on smoggy days than those under other weather conditions, indicating that airborne viruses could be tightly linked to human health in urban spaces. A previous study showed that in a children's daycare center, much more diverse and dominant human‐associated viruses were found in winter, while a high relative proportion and diversity of plant‐associated viruses were detected in summer, indicating that viral diversity was dependent on indoor air exchange with the outdoors air.^[^ ^26^ ^]^ In this study, a relatively high concentration of caudoviruses was observed on cloudy and smoggy days, similar to that found in the gut or animal viromes (Figure 6c). Air is not only a transport route for environmental microbes and respiratory viruses through aerosols, but also an ecosystem in which microbes circulate continuously.^[^ ^1^, ^2^, ^7^, ^48^ ^]^ This study, together with previous results, suggests an important channel for cross‐species transmission among viruses through the air that we breathe. This mode of transmission highlights the high frequency of transmission of DNA viruses in urban environments, which could have a great influence on the spread of diseases. Thus, the monitoring and management of air quality is important for ecological safety and public health. Although this study focused only on prokaryotic DNA viruses, the transmission mode of atmospheric viruses indicates the potential route for transmitting eukaryotic viruses that could pose a threat to animal health, including that of humans. Therefore, extensive and in‐depth researches on atmospheric DNA and RNA viruses in various environments are important.

## 4 Conclusion

We investigated airborne DNA viromes in urban spaces and discovered that the majority of viruses belonged to the dsDNA *Caudoviricetes* and ssDNA *Repensiviricetes* classes. Weather conditions can shape DNA viral communities, with the main biomarker of *Caudoviricetes* driven by air quality differences. We identified the genetic diversity of airborne viromes with novel candidate caudoviruses and genomoviruses at different taxonomic levels in the air. Airborne viruses carry diverse AMGs that are responsible mainly for the synthesis of nucleotide sugars, dNDP, dNTP and cofactors, as well as the degradation/utilization of sugars and amino acids, which could play crucial roles in viral DNA replication and survival in the atmosphere. Virus‐host linkages suggest that airborne viruses possibly infect *Campylobacterales* and *Enterobacterales*, particularly on smoggy days. A high prevalence of caudoviruses exists in the viromes of animal guts and polluted air, revealing the possibility of viral transmission between animals and urban spaces through aerosols on smoggy days. Taken together, this study illustrates the genetic diversity, ecological role and transmission mode of airborne viruses, which warrant further research in the future.

## 5 Experimental Section

### Sample Collection and Preparation

Air samples were collected in Beijing (116°23′E, 40°N) using the Bioaerosols and Dusts Sampling (BANDS) payload,^[^ ^46^ ^]^ including vacuum pump, gas flow detection device, sample collection box, solenoid valve, and air intake passage. The quartz fiber filter in a 0.2‐µm pore size was used to collect the bioaerosol particles with microbes, on the building terrace at an altitude of 55 m. The air intake components and exposed surface of the filters were sterilized before sampling. Microbes were collected on a piece of filter for 48 h, and three pieces of filters (6 days) were used as one of three samples for each weather condition (sunny, cloudy, rainy, LP and MP). An equal number of sterilized filters were used as the negative control. Weather type and meteorological parameter records were obtained from The China Weather Network (http://pc.weathercn.com). The weather division criteria are listed in Table S1 (Supporting Information). Each weather sampling event was replicated thrice (n = 3). However, owing to time constraints and weather quality, the study could only collect one MP sample that was grouped into smoggy days for statistical analysis. These filters were stored at −80 °C before nucleic acid extraction. Six‐day filters with a sufficient amount of biomass were combined as a sample to isolate virus like particle (VLP) and extract nucleic acids.

After grinding, the filter samples were washed with sterile buffer solution (0.2 m NaCl, 50 mm Tris‐HCl, 5 mm CaCl_2_, 5 mm MgCl_2_, pH 7.5) and subjected to three rounds of freeze‐thawing. Cell debris was removed using a 0.2‐µm ultrafiltration tube to remove eukaryotic and bacterial particles. To remove the interfering precipitate, the samples were centrifuged for 5 min at 4 °C at forces of 1 000 × *g*, 3 000 × g, 5 000 × *g*, 8 000 × *g*, 10 000 × *g*, and 12 000 × *g*, respectively. The supernatant was then transferred to an ultracentrifuge tube containing 28% (w/w) sucrose. After pre‐cooling on ice for 10 min, the samples were centrifuged by Himac CP 100WX Ultracentrifuge (Hitachi, Tokyo, Japan) at 300 000 × *g* for 2 h at 4 °C. The precipitate was suspended in buffer with 720 µL of water, 90 µL of 10 × DNase I buffer, 90 µL of 1 U/µL DNase I (TianGEN, Beijing, China), and 0.9 µL of 100 mg mL^−1^ RNase A (TianGEN^®^, Beijing, China). After shaking at 37 °C for 1 h, viral nucleic acid was extracted using Magen R6662‐02 MagPure Viral DNA/RNA Mini LQ Kit (MAGIGENE, Guangdong, China).

To overcome the scarcity of samples due to the extremely low density of viruses in air, whole‐genome amplification (WGA) was performed using the REPLI‐g Cell WGA & WTA Kit (Qiagen, Hilden, Germany), following the manufacturer's instructions. Sufficient nucleic acid amounts were obtained for library construction using 15‐min amplification to limit the biases of ssDNA. NanoDrop (Thermo Fisher Scientific, Waltham, USA), Qubit 4.0 (Life Technologies, CA, USA), and 1% agarose gel electrophoresis were used to detect the amplified products. The WGA nucleic acid amounts in different weather groups ranged from 5.63–6.72 µg, and there was no significant difference between groups (*p* > 0.05, ANOVA). The viral nucleic acid band size was ≈15 kb (Figure S1, Supporting Information). Qualified nucleic acids were stored at −80 °C and shipped to the MagiGene Technology Corporation (Guangdong, China) for metagenomic sequencing.

### Sequencing, Data Filter, Quality Control, and Read Assembly

Following the manufacturer's protocol, metagenome libraries of the qualified DNA products were constructed using the NEBNext Ultra II™ DNA Library Prep Kit for Illumina (New England Biolabs, MA, USA). The main steps were as follows: a) DNA sequence amplification and product fragmentation; b) terminal repair and 3′ terminal addition of polyA; c) adaptor connection, fragment selection, and purification; d) PCR amplification and purification. Qubit dsDNA HS Assay Kit (Life Technologies, CA, USA) and the Agilent 4200 TapeStation (Agilent Technologies, CA, USA) were used to qualify these libraries, followed by sequencing on Illumina Novaseq 6000 platform (Illumina, CA, USA) with 2 × 150 bp paired‐end read length. Quality control of the metagenomic sequencing data for each sample was performed to ensure the accuracy of the subsequent analysis. 263.5 Gb of raw reads were trimmed using Trimmomatic (v.0.36) with custom parameters (ILLUMINACLIP: TruSeq3‐PE. fa: 2:30:10 LEADING:3 TRAILING:3 SLIDINGWINDOW: 4:15 MINLEN:40).^[^ ^49^ ^]^ After removing adapters, polyX sequences, repeated reads, and low‐quality reads (Table S2, Supporting Information), 200.43 Gb clean reads were obtained and subjected further to removal of the viral host sequences using SOAPaligner^[^ ^50^ ^]^ and BWA^[^ ^51^ ^]^ (v.0.7.17, default parameter: mem‐k 30). The resulting high‐quality paired‐end reads for each viral sample were *de novo* assembled in contigs using MEGAHIT^[^ ^52^ ^]^ (v.1.1.2, default parameter: –presets meta‐large–min‐contig‐len 300) independently. All contigs were clustered using CD‐HIT^[^ ^53^ ^]^ (v.4.7, default parameter: ‐c 0.95 ‐aS 0.8) to obtain unique contigs (Table S3, Supporting Information).

### Sequence Filtering for Viral Identification and Classification Annotation

To obtain viral genes with high completeness, the assembled contigs were assessed and filtered by combining CheckV (v.0.7.0)^[^ ^54^ ^]^ and Visorter2 (v.2.2.4)^[^ ^55^ ^]^ using the default parameters. The Prodigal package encapsulated in CheckV software was used to annotate the assembled contigs, and the annotated genes of these fragments were compared with HMM models using seven major reference databases, including KEGG (https://www.kegg.jp/), VOGDB (https://www.vogdb.org/), PfamA/PfamB (http://pfam.xfam.org/), IMG/VR (https://img.jgi.doe.gov/vr/), TIGRFAM (www.tigr.org/TIGRFAMs) and RVDB (https://rvdb‐prot.pasteur.fr/). CheckV compares annotation information and GC content of two adjacent genes and determines the boundary between hosts and viruses according to the following conditions: a) the gene identification score must be greater than 1.2; b) when the sequence contains ten genes, the host or viral region were both determined by at least 2 microbial or viral genes; c) at least 30% of the genes in the host region are annotated as microbial genes. Notably, CheckV had three characteristic viral structures, including direct terminal repeats (DTR), inverted terminal repeats (ITR), and integrated provirus sequences, confirming the integrity of the viral sequences. Virsorer2 (v. 2.2.4) software was used to identify high‐confidence virus sequences based on their content and genomic structural characteristics, further confirming the results of CheckV and improving the sensitivity and accuracy of viral identification. 141 567 high‐confidence viral contigs was obtained accounting for 0.88% of all contigs (Table S3, Supporting Information).

After obtaining confident viral contigs with completeness >90%, VPF‐class (vpf‐tools 0.1.0.0 toolkit with default parameters)^[^ ^35^ ^]^ was used to compare the sequence with the viral protein family (IMG/VR database, https://img.jgi.doe.gov/vr/) and annotate the viral sequence taxonomic information. Using BWA v.0.7.17 software^[^ ^51^ ^]^ with default parameters (https://github.com/lh3/bwa/releases), the unique contigs were blasted against the virus‐NT database (v.2.9.0+) and the best hits (identity ≥ 80% and length of matched area ≥ 500 bp) with e ≤ 1e–5 were selected for species annotation. The annotations of VPF‐Class and CheckV were used to confirm viral taxonomy.^[^ ^35^, ^54^ ^]^ Viral sequences were classified according to the viral identification methods, confidence levels, and integrity information. The latest ICTV (International Committee on Taxonomy of Viruses) criteria were used to genetically and phylogenetically characterize each of the proposed novel taxonomies (https://ictv. global/).

### Viral Abundance and Diversity Analysis

By comparing the diversity and abundance of viral communities with contigs of different sequence sizes (Figure S16, Supporting Information), the confident viral contigs with sequence ≥ 1.5 kb were aligned to the obtained viral contigs using BWA (v. 0.7.17, default parameter: mem‐k 30) software,^[^ ^51^ ^]^ to filter the reads matched length <80% and calculate the RPKM (Reads Per Kilobase per Million mapped reads) value. The Alpha and Beta diversity metrics were computed based on the RPKM values of the viral sequences. Shannon index and Bray‐Curtis distance matrix were calculated to represent the viral diversity of each weather (alpha diversity) and the dissimilarity of viral composition between different weather condition (beta diversity), respectively.

LEfSe analysis was performed to find viral biomarkers with statistical differences using the OmicStudio tool (https://www.omicstudio.cn/tool/). First, a non‐parametric Kruskal‐Wallis H test was used to detect contigs with significant differences in abundances between different groups. The Wilcoxon rank‐sum test was then used to test the differences in the consistency of the contigs between subgroups in the previous step. Finally, LDA (linear discriminant analysis) was used to estimate the effect of each contig abundance on the difference (LDA > 2.0 and *p* < 0.05).

These three analyses were based on the RPKM value of the virus sequences, and the formula was as follows:

(1) $$\mathrm{RPKM}=\frac{\mathrm{Contig}\;\mathrm{reads}}{\mathrm{Total}\;\mathrm{mapped}\;\mathrm{reads}\;\left(\mathrm{millions}\right)\;\times \;\mathrm{Contig}\;\mathrm{length}\;\left(\mathrm{KB}\right)}$$

where contig reads are the number of reads mapped to each contig. The total number of mapped reads are the total number of reads blasted to the viral contigs. Contig length is the length of the contig.

### Host Prediction, Phylogenetic Analysis, and Genome Annotation

SpacePHARER (CRISPR Spacer Phage–Host Pair Finder) software (v2.fc5e668) with a sensitivity of 7.5 (“‐s7.5”)^[^ ^56^ ^]^ and VPF‐Class based on the viral protein families^[^ ^35^ ^]^ was used to predict and analyze phage‐host relationships. Information on the host species of the bacteriophages was identified using bacteriophage bases that matched the CRISPR spacer regions in the genomic or metagenomic data. Specifically, an optimal match between the virus and host was obtained by comparing the CRISPR spacer and phage at the protein level. Species information on the host at seven levels (domain, phylum, class, order, family, genus, and species) was determined by controlling for false positives, based on the dataset and by improving identification accuracy.

To identify potential new viruses, high‐confidence viral contigs were chosen based on the completeness and length of the sequences. These contigs were further uploaded to VIPTree^[^ ^57^ ^]^ to construct a phylogenetic tree based on genome‐wide sequence similarities computed using tBLASTx (https://www.genome.jp/viptree/). An entire proteomic tree was constructed by comparing airborne viruses with all the reference viral genomes. Viral genomes closest to the sequences of airborne viruses were selected as references. Finally, the focused proteomic trees were regenerated using airborne viruses and references. These viral contigs with complete genomes, but without defined families, genera, or species, were selected to identify new viruses by constructing phylogenetic trees. An evolutionary tree based on the viral core gene was constructed by searching protein sequences against the HMM database using hmmsearch with e < 1e–5.^[^ ^58^, ^59^ ^]^ The BLAST searches against the NCBI nr database were used to check matches. The retrieved protein sequences were aligned using MEGA 11^[^ ^60^ ^]^ and a tree was built using IQ‐TREE (v.2.3.4) with 1000 ultrafast bootstraps.^[^ ^61^ ^]^

Potential promoter regions in the genome sequence were determined using the Neural Network Promoter Prediction program of the Berkeley Drosophila Genome Project (NNPP v.2.2, cutoff scores of 0.90 and 0.95) with prokaryotic organisms (http://www.fruitfly.org/seq_tools/promoter.html). Viral genome information, including tail, head, and packaging, was analyzed using the scalable bacteriophage annotation tool Pharokka^[^ ^62^ ^]^ and further annotated by comparison with viral information from the UniProtKB/Swiss‐Prot database (ViralZone, reviewed protein, https://viralzone.expasy.org) with a threshold of e < 1e‐3. Conversed domains were searched in Pfam and their structures were constructed using trRosetta2 software (v.1.13).^[^ ^63^ ^]^ All protein structures were analyzed using VMD 1.9.4 (Visual Molecular Dynamics, https://www.ks.uiuc.edu/Research/vmd/). The KEGG database (https://www.kegg.jp/) was used to analyze gene functions (e ≤ 1e–3) and biological systems, such as cells, organisms and ecosystems. The ORFs from viral confident contigs (1.5–184.2 kb) were imported into HMMScan (e < 1e–5 and bit score >50) for further annotation from the pfam database (http://pfam.xfam.org/).^[^ ^58^ ^]^ Gene positions and matched sequences (≥100 bp) were manually checked in viral contigs to validate the functional annotation of putative AMGs. Finally, putative AMGs were compared with viral AMG data derived from previous studies.^[^ ^27^, ^33^ ^]^

### Data and Materials Availability

The sequencing data of all 13 samples from the viral metagenomic libraries have been deposited in the NCBI Sequence Read Archive (SRA) under Bioproject ID PRJNA854686 with accession no. SRR19981422 to SRR19981434. All data required to evaluate the conclusions of this paper are presented in the paper and/or Supplementary Materials. Additional data related to this study may be requested from the authors.

### Statistical Analysis

All measurements were performed at least in triplicate. Data are presented as mean ± SEM, n ≥ 3. One‐way ANOVA with Scheffé’s test was used to identify samples with differences between multiple groups. The Kruskal‐Wallis H test with Dunn's test was used to analyze significant differences between the two groups. The Wilcoxon rank‐sum test was used to test for differences in consistency between groups. Statistical analysis was performed to calculate *p*‐values in Python using DataFrame SciPy v.1.13.1.^[^ ^64^ ^]^ In all cases, significant differences were defined as *(*p* < 0.05), **(*p* < 0.01) and ***(*p* < 0.001).

## Conflict of Interest

The authors declare no conflict of interest.

## Author Contributions

A.D. and T.W. conceived and designed the study. J.W. and A.D. performed the experiments and all data analysis with assistance from R.S., L.L., and X.L. A.D. and J.W. wrote the manuscript with the revision from T.W. All of the authors have approved the final paper.

## Supporting information



Supporting Information

## Data Availability

The data that support the findings of this study are available from NCBI Sequence Read Archive (SRA). Restrictions apply to the availability of these data, which were used under license for this study. Data are available from the authors with the permission of NCBI Sequence Read Archive (SRA).
