# Supplementary material for: Synoptic Variation Drives Genetic Diversity and Transmission Mode of Airborne DNA Viruses in Urban Space
Source: Adv Sci (Weinh). 2024 Oct 22;11(46):2404512. doi: 10.1002/advs.202404512 (PMC11633480; doi:10.1002/advs.202404512)
Supplement: Supplementary file 1 — Supporting Information [file ADVS-11-2404512-s001.pdf]

ADVANCED SCIENCE
Open Access

Supporting Information

for *Adv. Sci.*, DOI 10.1002/advs.202404512

Synoptic Variation Drives Genetic Diversity and Transmission Mode of Airborne DNA Viruses in Urban Space

Aihua Deng, Junyue Wang, Lai Li, Ruilin Shi, Xuemin Li and Tingyi Wen**

Supplementary Materials

Synoptic Variation Drives Genetic Diversity and Transmission Mode of Airborne DNA Viruses in Urban Space

Aihua Deng[1,2,3†]*, Junyue Wang[2†], Lai Li[2], Ruilin Shi[2], Xuemin Li[4], Tingyi Wen[2,5]*

[1] State Key Laboratory of Animal Nutrition and Feeding, Ministry of Agriculture and Rural Affairs Feed Industry Centre, China Agricultural University, Beijing 100193, RP China

[2] State Key Laboratory of Microbial Resources, Institute of Microbiology, Chinese Academy of Sciences, Beijing 100101, PR China

[3] Beijing Bio-Feed Additives Key Laboratory, Beijing 100193, PR China

[4] Department of Ophthalmology, Peking University Third hospital, Beijing 100191, China

[5] Savaid medical school, University of Chinese Academy of Sciences, Beijing, 100049, PR China

*Correspondence should be addressed to A.D. (dengah@cau.edu.cn) and T.W. (wenty@im.ac.cn)

[†] These authors contributed equally to this work.

Short title: Diversity and transmission of airborne viruses

Table S1 Sample collection information and the recorded meteorological parameters.

Sample	Date	RH (%)	T (℃)	AQI	AQI level	Weather division criteria
Sunny-1	30-Jul-21	79	22-27	16	I-II	There are no clouds in the sky, or scattered clouds, but the low to medium cloud amount is less than 10%. High cloud amount is less than 40%.
	31-Jul-21	72	23-34	67		
	1-Aug-21	39	22-32	22		
	2-Aug-21	55	23-31	17		
	6-Aug-21	61	22-32	18		
	7-Aug-21	55	23-32	90		
Sunny-2	9-Aug-21	77	22-31	33	I-II	
	10-Aug-21	77	23-31	19		
	21-Aug-21	37	22-31	41		
	22-Aug-21	68	23-31	70		
	25-Aug-21	52	20-27	19		
	26-Aug-21	39	18-30	22		
Sunny-3	29-Sep-21	13	18-24	58	I-II	
	7-Oct-21	59	8-18	14		
	8-Oct-21	51	12-20	44		
	11-Oct-21	52	6-18	30		
	12-Oct-21	78	10-19	50		
	13-Oct-21	77	12-18	24		

Continue

Sample	Date	RH (%)	T (℃)	AQI	AQI level	Weather division criteria
Cloudy-1	3-Aug-21	70	24-31	39	I-II	There are 40% to 100% percent of clouds in the sky.
	4-Aug-21	82	24-32	35		
	5-Aug-21	73	23-30	26		
	11-Aug-21	70	22-31	52		
	12-Aug-21	79	22-29	30		
	13-Aug-21	86	21-29	43		
Cloudy-2	14-Aug-21	66	22-28	38	I-II	
	29-Aug-21	61	20-28	58		
	30-Aug-21	77	20-28	66		
	28-Sep-21	86	18-24	59		
	30-Sep-21	77	16-22	49		
	2-Oct-21	78	19-20	61		
Cloudy-3	1-Nov-21	73	5-15	80	II	
	2-Nov-21	75	6-13	74		
	6-Nov-21	98	0-13	66		
	7-Nov-21	89	-1-1	96		
	15-Nov-21	52	-1-12	60		
	16-Nov-21	58	2-12	99		

Continue

Sample	Date	RH (%)	T (℃)	AQI	AQI level	Weather division criteria
Rainy-1	15-Aug-21	65	22-28	32	I-II	Evidently, there is rain falling.
	16-Aug-21	82	23-28	51		
	17-Aug-21	45	20-29	32		
	18-Aug-21	71	21-30	23		
	19-Aug-21	94	21-26	30		
	20-Aug-21	61	21-28	37		
Rainy-2	23-Aug-21	59	20-30	48	I	
	24-Aug-21	72	19-28	19		
	27-Aug-21	54	17-29	14		
	28-Aug-21	43	18-27	23		
	24-Sep-21	95	16-23	27		
	25-Sep-21	63	19-25	30		
Rainy-3	26-Sep-21	91	17-22	16	I	
	27-Sep-21	51	17-27	42		
	13-Oct-21	77	12-18	24		
	14-Oct-21	65	12-21	32		
	15-Oct-21	19	6-20	41		
	6-Nov-21	98	0-13	42		

**Sample**	**Date**	**RH (%)**	**T (°C)**	**AQI**	**AQI level**	**Weather division criteria**
LP-1	28-Oct-21	73	7-16	129	III	The AQI index is 101-150.
	29-Oct-21	45	7-18	118		
	30-Oct-21	55	9-21	154		
	31-Oct-21	73	8-14	127		
LP-2	4-Nov-21	82	7-18	139	III	
	5-Nov-21	89	11-15	143		
	17-Nov-21	76	2-12	147		
	18-Nov-21	90	1-9	132		
LP-3	28-Nov-21	81	3-11	146	III	
	29-Nov-21	65	-2-5	101		
	9-Jan-22	59	-6-3	124		
	10-Jan-22	68	-9-2	113		
MP	26-Mar-20	82	10-15	199	IV	The AQI index is 151-200.
	27-Mar-20	62	11-20	182		

RH, relative humidity; T, temperature; AQI, air quality index; LP, Light pollution; MP, moderate pollution; I, the AQI index of 1-50; II, the AQI index of 51-100; III, the AQI index of 101-150; IV, the AQI index of 151-200. The records of weather types and meteorological parameters were referred from The China Weather Network (http://pc.weathercn.com).

Table S2 Overview of the viral sequences for air samples.

Sample	WGA (µg)	Raw reads	Clean reads	Virus reads	Virus ratio (%)
Sunny-1	5.14	89174665	77428803	3746726	4.84
Sunny-2	5.54	72592756	57348554	5991493	10.45
Sunny-3	7.20	66528754	55796558	3731787	6.69
Cloudy-1	7.58	79103440	67801633	4004433	5.91
Cloudy-2	7.01	72960009	60837019	4550973	7.48
Cloudy-3	5.59	72039198	30217424	688250	2.28
Rainy-1	3.62	75498519	59138659	12268031	20.74
Rainy-2	8.09	65599831	54576599	4203279	7.70
Rainy-3	7.03	70172858	58338709	3649843	6.26
LP-1	6.43	43375522	35073243	1001525	2.86
LP-2	5.83	38950453	31116610	1046632	3.36
LP-3	4.63	48685149	38365689	966369	2.52
MP	5.73	83474998	73394431	2760932	3.76

Sample, weather types; WGA, Total amount of viral nucleic acid amplified from the whole genome; Raw reads, original reads; Clean reads, the number of high-quality reads after removing adapters and low-quality reads; Virus reads, the number of PE reads of clean reads blasted to viral contigs; Virus ratio (%), the ratio of Virus reads/Clean reads.

Table S3 Contigs assembly of sunny, cloudy, rainy and smoggy (LP and MP) viruses.

Sample	Total num	Max len	Min len	N50	GC (%)
Sunny-1	605125	233395	300	1407	36.96
Sunny-2	1289701	21234	300	622	39.99
Sunny-3	1969925	224956	300	741	45.78
Cloudy-1	1749049	286627	300	808	30.55
Cloudy-2	2129949	333412	300	746	47.78
Cloudy-3	27298	32338	300	663	55.34
Rainy-1	340372	233424	300	989	38.68
Rainy-2	2330345	48148	300	611	43.01
Rainy-3	1822703	208766	300	978	49.2
LP-1	838018	34086	300	713	55.51
LP-2	74084	15741	300	720	53.68
LP-3	254637	25000	300	745	57.85
MP	2590393	561001	300	575	39.2
Total contigs	16021599	561001	300	794	45.66
Confidence viral contigs	141567	184229	300	1169	45.86

Sample, weather types; LP, Light pollution; MP, moderate pollution; Total num, the total number of contigs obtained by the assembly; Max len, the maximum length of contig obtained by the assembly; Min len, minimum length of contig obtained by the assembly; N50, the contigs are arranged in the order from largest to smallest. When the length reaches 50% of all contigs, the length of the contig is contig N50. The larger N50 is, the higher the quality of genome assembly is. GC, regions with the GC content of about 50% on the genome are easier to be detected, resulting in more reads and higher coverage. Regions with high GC or low GC are not easy to be detected, resulting in fewer reads and lower coverage of these regions. Confidence viral contigs: viral contigs with high, medium and low completeness are identified by CheckV and Visorter2, accounting for 0.88% in all contigs.

Table S4 Presence of dsDNA and ssDNA viral orders in different weathers.

	Sunny	Cloudy	Rainy	LP	MP
dsDNA	*Priklausovirales*	*Cryppavirales*	*Cryppavirales*	*Rowavirales*	*Kalamavirales*
	Cryppavirales	*Rowavirales*	*Rowavirales*	*Asfuvirales*	*Cryppavirales*
	Rowavirales	*Priklausovirales*	*Kalamavirales*	*Herpesvirales*	*Rowavirales*
	Asfuvirales	*Kalamavirales*	*Priklausovirales*	*Chitovirales*	*Ortervirales*
	Kalamavirales	*Asfuvirales*	*Asfuvirales*	*Ortervirales*	*Asfuvirales*
	Herpesvirales	*Herpesvirales*	*Ortervirales*	*Pimascovirales*	*Chitovirales*
	Chitovirales	*Ortervirales*	*Herpesvirales*	*Cryppavirales*	*Herpesvirales*
	Pimascovirales	*Pimascovirales*	*Imitervirales*	*Kalamavirales*	*Pimascovirales*
	Ortervirales	*Chitovirales*	*Pimascovirales*	*Algavirales*	*Imitervirales*
	Imitervirales	*Imitervirales*	*Chitovirales*	*Imitervirales*	*Algavirales*
	Algavirales	*Algavirales*	*Algavirales*		
	Unclassfied caudoviruses *(Caudoviricetes)*	Unclassfied caudoviruses (*Caudoviricetes*)	Unclassfied caudoviruses *(Caudoviricetes)*	Unclassfied caudoviruses (*Caudoviricetes*)	Unclassfied caudoviruses (*Caudoviricetes*)
ssDNA	*Polivirales*	*Piccovirales*	*Cirlivirales*	*Cirlivirales*	*Petitvirales*
	Piccovirales	*Zurhausenvirales*	*Petitvirales*	*Geplafuvirales*	*Geplafuvirales*
	Cirlivirales	*Petitvirales*	*Mulpavirales*	*Petitvirales*	*Tubulavirales*
	Petitvirales	*Cirlivirales*	*Tubulavirales*	*Tubulavirales*	
	Mulpavirales	*Mulpavirales*	*Geplafuvirales*		
	Tubulavirales	*Tubulavirales*			
	Geplafuvirales	*Geplafuvirales*			

Table S5 Domain information of hypothetical proteins.

Gene number	Description	Identity (%)	E-value
**AirCV1**			
CDS_0017	Virion structural protein containing putativePKD domain, DUF2793 domain	48.3	6.00E-124
CDS_0023	Tail tubular protein A	54.4	5.10E-77
CDS_0025	Bacteriophage lambda head decoration protein D	57.9	2.00E-52
CDS_0026	N4-gp56 family major capsid protein	67.4	0
CDS_0040	Putative endodeoxyribonuclease I containing DUF1064 domain	52.7	1.20E-34
CDS_0043	Terminase small subunit	56.8	3.50E-80
CDS_0047	DNA-binding protein for Bacteriophage lambda Replication	74.8	2.40E-168
CDS_0056	Bacteriophage lambda Replication protein O N-terminal domain	41.7	1.30E-32
CDS_0057	Phage antirepressor protein YoqD, KilAC domain	45.3	4.20E-52
CDS_0058	Transcriptional regulator containing HTH cro/C1-type domain	45.9	1.80E-25
CDS_0059	YdaS antitoxin of YdaST toxin-antitoxin system	54.7	2.00E-13
CDS_0060	Peptidase S24 containing HTH cro/C1-type domain	76.7	1.30E-89
CDS_0074	Putative excisionase containing HTH deoR-type domain	62.7	2.40E-23
**AirCV2**			
CDS_0001	Helicase-like protein	33.9	1.40E-71
CDS_0006	Glutamine--fructose-6-phosphate aminotransferase	25.4	1.5
CDS_0011	Putative lysozyme domain protein	40.5	1.90E-34
CDS_0023	DNA-invertase hin	48.9	1.20E-61
CDS_0025	Plasmid segregation oscillating ATPase ParF	36.0	3.20E-34
CDS_0028	Phage repressor protein C	26.0	4.60E-14
CDS_0031	DNA replication protein DnaC	28.6	4.70E-15

CDS_0034	XRE family transcriptional regulator	28.8	8.90E-04
CDS_0061	HNH endonuclease	41.5	2.90E-06
CDS_0084	baseplate protein	40.0	3.50E+00
CDS_0089	Tail sheath protein	29.9	7.30E-14
CDS_0091	Defence against restriction A N-terminal domain-containing protein	29.0	1.70E-06
CDS_0094	Baseplate protein J-like domain-containing protein	37.1	1.30E-83
CDS_0096	Phage protein	23.5	1.10E-06
CDS_0098	Tail protein	28.2	4.20E-28
CDS_0099	Phage baseplate assembly protein V	37.4	2.80E-14
CDS_0101	Phage portal protein	32.3	3.60E-72
CDS_0109	Toxin-antitoxin system, HicB family	48.9	2.60E-33
CDS_0110	DNA methyltransferase with helicase C-terminal domain-containing protein	37.8	1.20E-85
**AirCV3**			
CDS_0013	HNH endonuclease	41.5	2.90E-06
CDS_0039	XRE family transcriptional regulator	28.8	8.90E-04
CDS_0042	DNA replication protein DnaC	28.6	4.70E-15
CDS_0045	Phage repressor protein C	26.0	4.60E-14
CDS_0048	Plasmid segregation oscillating ATPase ParF	36.0	3.20E-34
CDS_0050	DNA-invertase hin	48.9	1.20E-61
CDS_0067	Glutamine--fructose-6-phosphate aminotransferase	25.4	1.5
CDS_0072	Helicase-like protein	35.0	0
CDS_0073	Toxin-antitoxin system, HicB family	48.9	2.60E-33
CDS_0074	Type II toxin-antitoxin system HicA family toxin	62.7	1.90E-15
CDS_0081	Phage portal protein	32.3	3.60E-72
CDS_0083	Phage baseplate assembly protein V	37.4	2.80E-14
CDS_0084	Tail protein	28.2	4.20E-28
CDS_0086	Phage protein	23.5	1.10E-06
CDS_0088	Baseplate protein J-like domain-containing protein	37.1	1.30E-83
CDS_0091	Defence against restriction A N-terminal domain-containing protein	29.0	1.70E-06
CDS_0093	Tail sheath protein	29.9	7.30E-14

CDS_0098	Baseplate protein	40.0	3.50E+00

Table S6 The antimicrobial resistance gene (ARG) information of airborne viruses.

**Contig**	**Gene_ID**	**Function**	**ARG class**	**Identity (%)**	**E-value**
Cloudy.1_contig_4285	NIFDJALF_29019	putative transcriptional regulator; K08365 MerR family transcriptional regulator, mercuric resistance operon regulatory protein (A)	MerR	62	4.06E-48
Cloudy.2_contig_1039492	NIFDJALF_42211	benomyl/methotrexate resistance protein; K08158 MFS transporter, DHA1 family, multidrug resistance protein (A)	MDR1	25.5	5.45E-10
Cloudy.2_contig_1087236	NIFDJALF_42508	Bcr/CflA subfamily Drug resistance transporter; K07552 MFS transporter, DHA1 family, bicyclomycin/chloramphenicol resistance protein (A)	Bcr/CflA	31.2	3.97E-10
Cloudy.2_contig_136205	NIFDJALF_34639	hypothetical protein; K03327 multidrug resistance protein, MATE family (A)	Tc	86.1	0
Cloudy.2_contig_458747	NIFDJALF_37837	hypothetical protein; K08157 MFS transporter, DHA1 family, multidrug resistance protein (A)	TPO1	34.2	2.80E-20
Cloudy.2_contig_726318	NIFDJALF_40098	hypothetical protein; K08157 MFS transporter, DHA1 family, multidrug resistance protein (A)	TPO1	27.1	1.53E-09
Cloudy.2_contig_756887	NIFDJALF_40261	hypothetical protein; K08157 MFS transporter, DHA1 family, multidrug resistance protein (A)	TPO1	26.5	5.89E-17
Cloudy.2_contig_875788	NIFDJALF_41153	hypothetical protein; K08158 MFS transporter, DHA1 family, multidrug resistance protein (A)	MDR1	88.8	3.74E-107
Cloudy.2_contig_91348	NIFDJALF_34220	hypothetical protein; K08157 MFS transporter, DHA1 family, multidrug resistance protein (A)	TPO1	32.1	5.63E-12
Cloudy.2_contig_91348	NIFDJALF_34221	KAFR0F04390; hypothetical protein; K08157 MFS transporter,	TPO1	27.3	7.39E-12

		DHA1 family, multidrug resistance protein (A)			
Cloudy.2_contig_91407	NIFDJALF_34247	hypothetical protein; K03327 multidrug resistance protein, MATE family (A)	Tc	83.3	0
MP.2_contig_39614	NIFDJALF_100842	probable multidrug resistance protein; K03446 MFS transporter, DHA2 family, multidrug resistance protein (A)	EmrB	69.6	1.98E-57
Rainy.1_contig_219076	NIFDJALF_53377	emrB; multidrug resistance protein B; K03446 MFS transporter, DHA2 family, multidrug resistance protein (A)	EmrB	100	0
Rainy.3_contig_1435967	NIFDJALF_82104	hypothetical protein; K08157 MFS transporter, DHA1 family, multidrug resistance protein (A)	TPO1	70.3	1.64E-21
Rainy.3_contig_1435967	NIFDJALF_82106	hypothetical protein; K08157 MFS transporter, DHA1 family, multidrug resistance protein (A)	TPO1	91.2	6.71E-132
Rainy.3_contig_1435967	NIFDJALF_82105	hypothetical protein; K08157 MFS transporter, DHA1 family, multidrug resistance protein (A)	TPO1	74.6	1.19E-27
Rainy.3_contig_1435967	NIFDJALF_82107	hypothetical protein; K08157 MFS transporter, DHA1 family, multidrug resistance protein (A)	TPO1	74.5	2.99E-17
Rainy.3_contig_1435967	NIFDJALF_82108	hypothetical protein; K08157 MFS transporter, DHA1 family, multidrug resistance protein (A)	TPO1	64.6	1.83E-37
Rainy.3_contig_1453721	NIFDJALF_82226	hypothetical protein; K08158 MFS transporter, DHA1 family, multidrug resistance protein (A)	MDR1	33.8	9.92E-08
Rainy.3_contig_1501918	NIFDJALF_82697	Inner membrane component of tripartite multidrug resistance system; K03446 MFS transporter, DHA2 family, multidrug resistance protein (A)	EmrB	34.1	4.86E-06
Rainy.3_contig_300362	NIFDJALF_70035	Major facilitator superfamily transporter; K08158 MFS transporter, DHA1 family, multidrug resistance protein (A)	MDR1	38.7	1.45E-32
Rainy.3_contig_546166	NIFDJALF_73180	hypothetical protein; K18443 golgi-specific brefeldin A-resistance guanine nucleotide exchange factor 1 (A)	GBF1	39.7	3.42E-06

Rainy.3_contig_740723	NIFDJALF_75266	hypothetical protein; K08158 MFS transporter, DHA1 family, multidrug resistance protein (A)	MDR1	90.6	1.58E-140
Rainy.3_contig_740723	NIFDJALF_75269	hypothetical protein; K08158 MFS transporter, DHA1 family, multidrug resistance protein (A)	MDR1	77.5	1.25E-55
Rainy.3_contig_740723	NIFDJALF_75271	hypothetical protein; K08158 MFS transporter, DHA1 family, multidrug resistance protein (A)	MDR1	75.3	1.57E-111
Rainy.3_contig_740723	NIFDJALF_75268	hypothetical protein; K08158 MFS transporter, DHA1 family, multidrug resistance protein (A)	MDR1	82.5	3.05E-24
Smoggy_contig_2251101	NIFDJALF_118458	putative heavy metal transcriptional regulator; K08365 MerR family transcriptional regulator, mercuric resistance operon regulatory protein (A)	MerR	98.7	2.55E-47
Sunny.3_contig_1807980	NIFDJALF_27958	hypothetical protein; K08157 MFS transporter, DHA1 family, multidrug resistance protein (A)	TPO1	26.8	5.31E-11
Sunny.3_contig_404702	NIFDJALF_18343	hypothetical protein; K03327 multidrug resistance protein, MATE family (A)	Tc	70.5	4.49E-32
Sunny.3_contig_427498	NIFDJALF_18577	hypothetical protein; K08158 MFS transporter, DHA1 family, multidrug resistance protein (A)	MDR1	32.9	1.52E-67

Table S7 Overview of the eukaryotic hosts for air viral samples.

Domain	Host classification	Abundant	Viruses
Eukaryota	P__*Streptophyta*.C__*Magnoliopsida*.O__*Malpighiales*.F__*Euphorbiaceae*.G__*Manihot*	0.01%	*Caulimoviridae*
	P__*Streptophyta*.C__*Magnoliopsida*.O__*Fabales*.F__*Fabaceae*.G__*Glycine*	1.93%	*Caulimoviridae*
	P__*Chordata*.C__*Mammalia*.O__*Primates*.F__*Hominidae*.G__*Homo*	0.06%	*Baikalvirus*<br>*Bracovirus*<br>*Sputnikvirus*<br>*Ichnovirus*

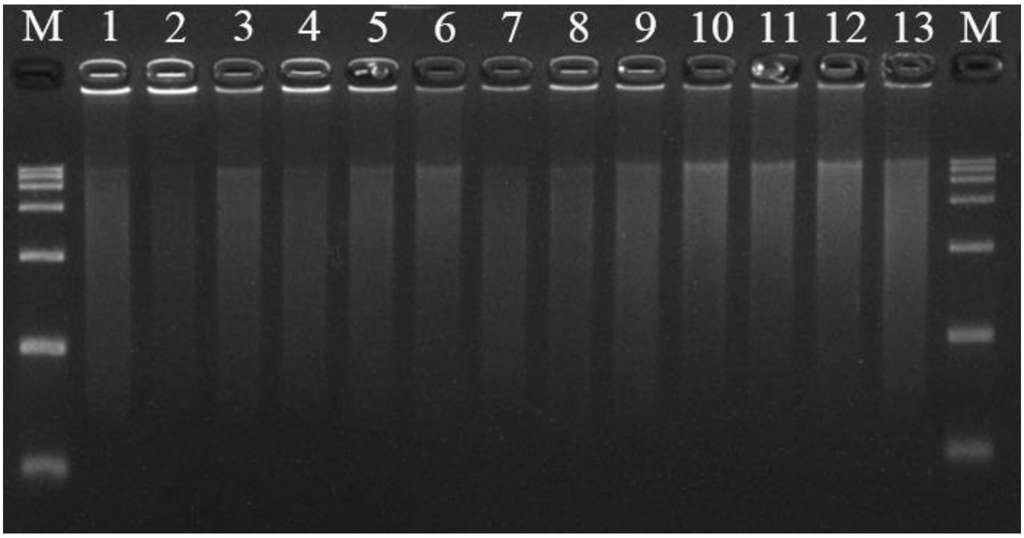

Figure S1 Agarose gel electrophoresis of nucleic acid by whole-genome amplification (WGA). 1-3, Sunny; 4-6, Cloudy; 7-9, Rainy; 10-12, LP; 13, MP; M, DL15000. 1% agarose gel electrophoresis were used to detect the amplified products. The WGA nucleic acid amounts in different weather groups ranged from 5.63–6.72 μg, and there was no significant difference between groups ($p > 0.05$, ANOVA).

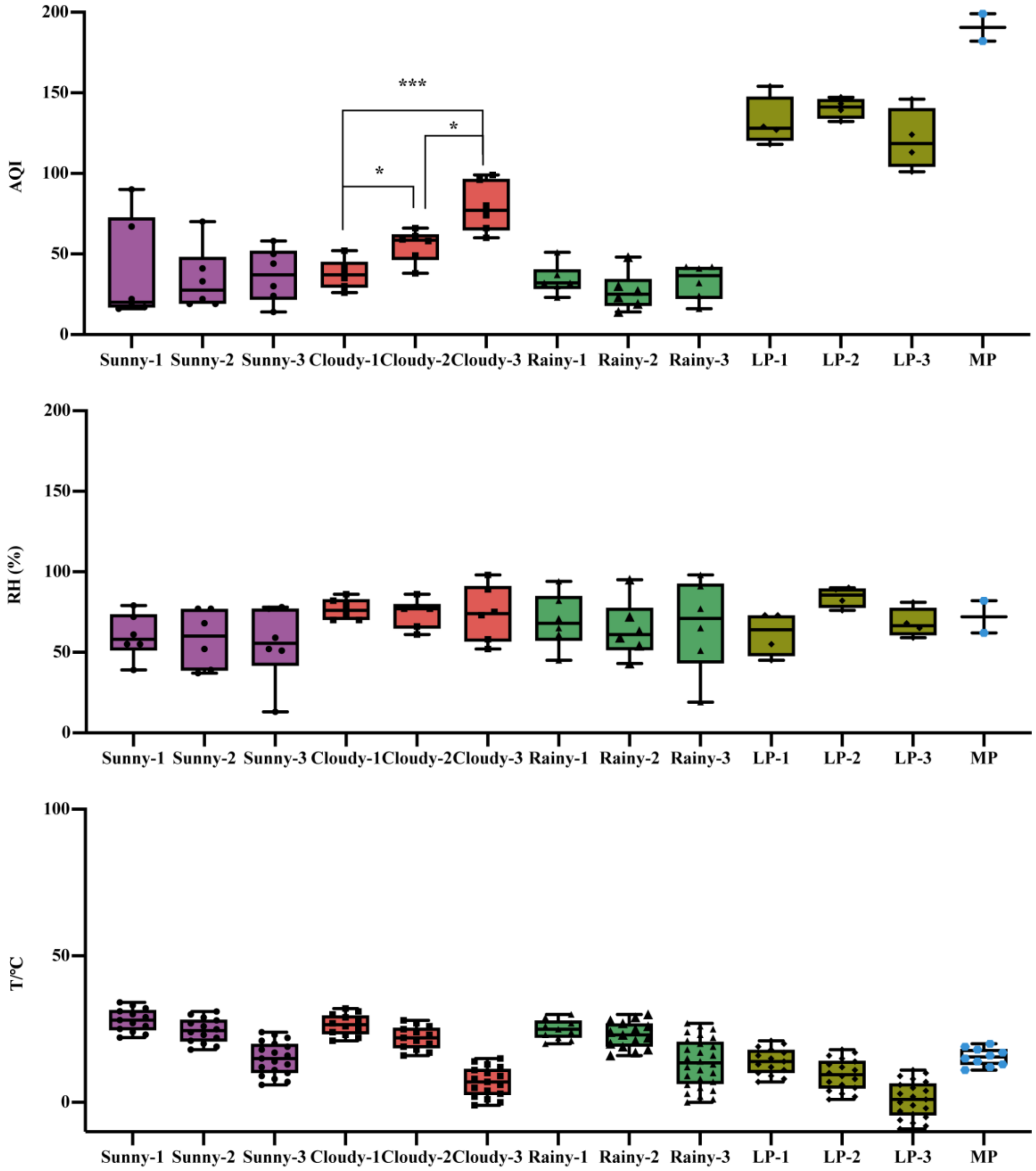

Figure S2 The meteorological parameter record of samples. There was no significant difference between different kinds of weather samples except for the AQI record of cloudy samples. The AQI value of Cloudy-3 was significantly higher than those of the other two samples in the cloudy group, suggesting that the air quality of Cloudy-3 was closer to light air pollution. *, $p < 0.05$; ***, $p < 0.001$.

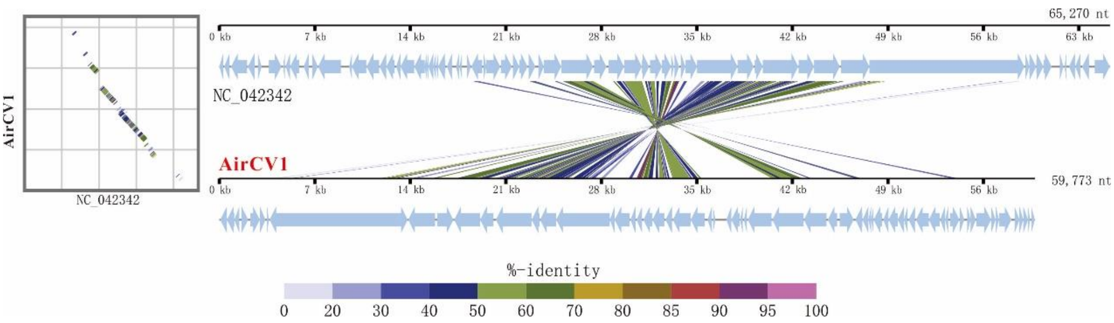

Figure S3 The genomic alignment of AirCV1 with the closest reference *Hollowayvirus* (NC_042342). Pairwise dot plots of these genomes are shown. Colored lines in the alignment and the dot plots indicate tBLASTx results (e-value < 1e-2). Grid lines in the dot plots indicate 40 kb intervals.

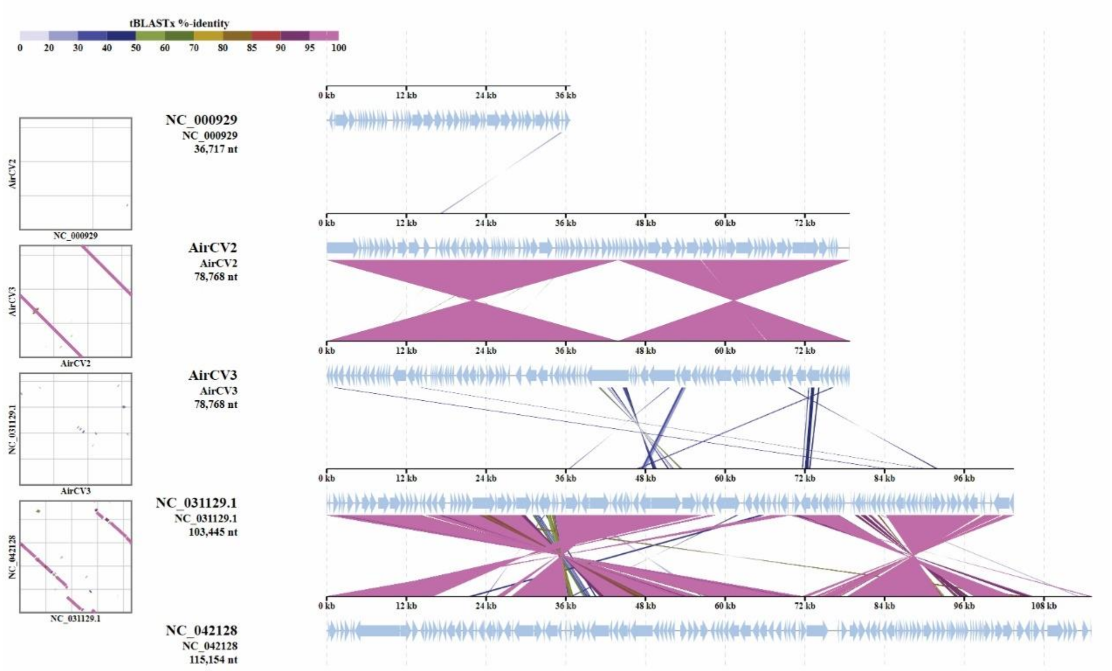

Figure S4 Genomic alignments of AirCV2 and AirCV3 with the closest references *Muvirus* (NC_000929) and *Punavirus* (NC_031129.1 and NC_0421128). AirCV2 clustered with AirCV3, sharing >95% sequence identity. Their alignment identities with *Muvirus* and *Punavirus* were lower than 40%.

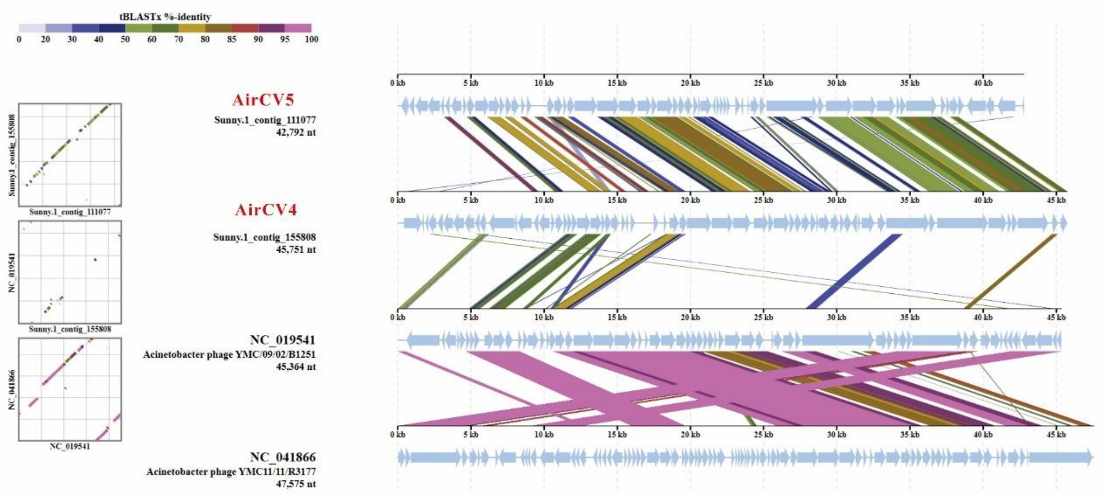

Figure S5 Genomic alignments of AirCV4 and AirCV5 with the closest references *Acinetobacter* phage (NC_019541 and NC_041866). AirCV4 clustered with AirCV5, forming a clade close to the *Acinetobacter* phage, with 4–7% coverage for similar sequences.

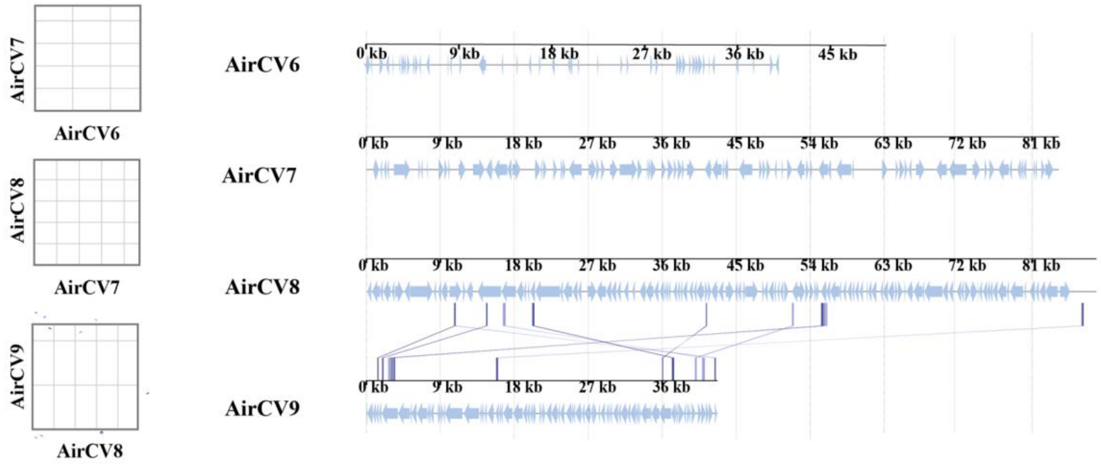

Figure S6 Genomic alignments of AirCVs 6-9. As shown in the pairwise dot plots of these genomes, no sequence was aligned to the known references and their similarities to each other were extremely low.

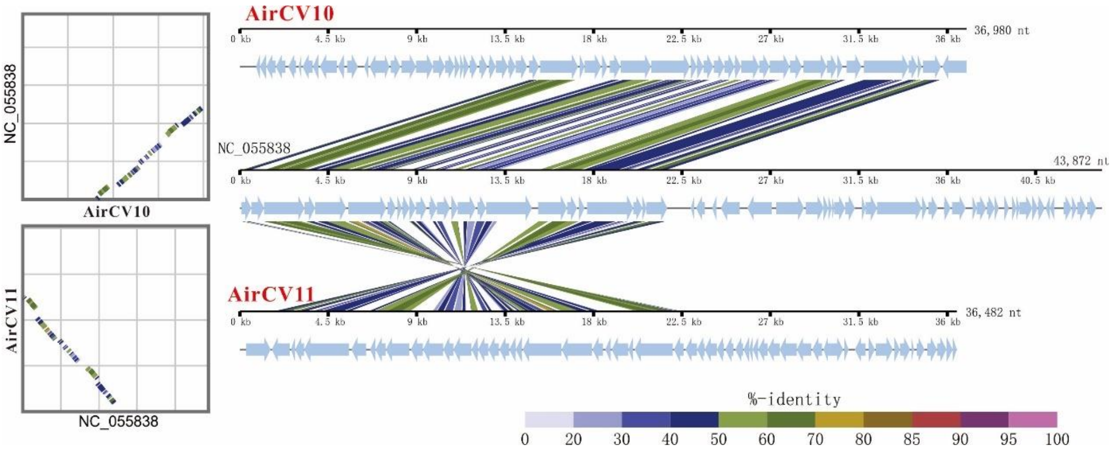

Figure S7 Genomic alignments of AirCV10 and AirCV11 with the closest reference *Xanthomonas* phage (NC_055838). AirCV10 clustered with AirCV11, sharing > 95% identity and therefore belonging to the same taxonomy. They had low alignment with *Xanthomonas* phage because nearly half of the contigs were unmatched.

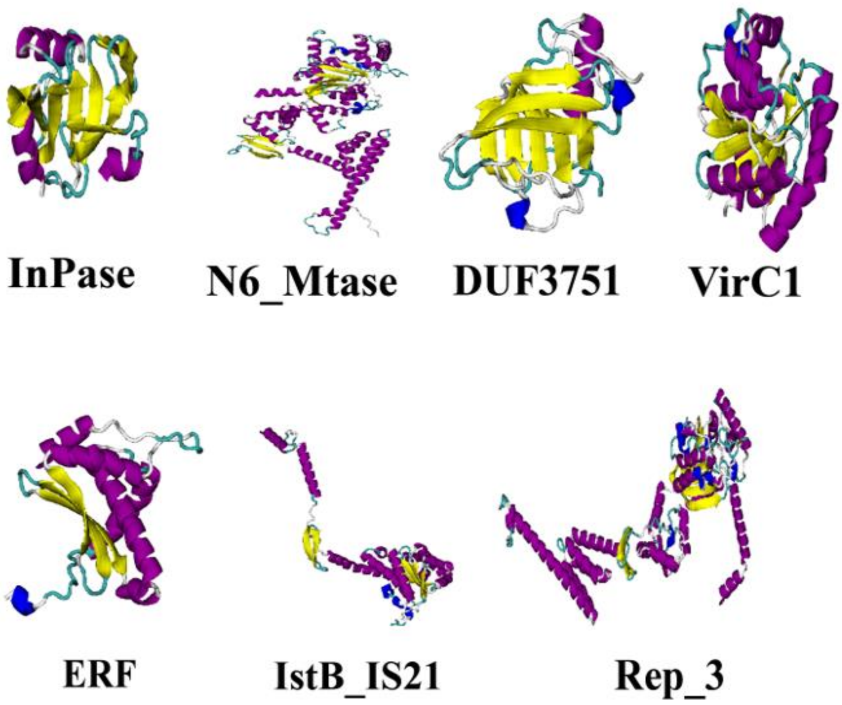

Figure S8. Domain structures of predicted proteins of AirCVs 2 and 3. AirCV2 has a specific N-6 DNA Methylase (N6_Mtase, CL0063), while other domains were as same

as AirCV3 including inorganic pyrophosphatase (InPase), initiator replication protein (Rep_3, CL0123), VirC1 protein (VirC1, CL0023), ERF superfamily (ERF), IstB-like ATP binding protein (IstB_IS21, CL0023), and phage tail-collar fibre protein (DUF3751). The coloring method was Secondary Structure and the drawing method was NewCartoon. α-helix shows in purple, β-pleated shows in yellow, β-turn shows in cyan, random coil shows in white and other helixes show in blue.

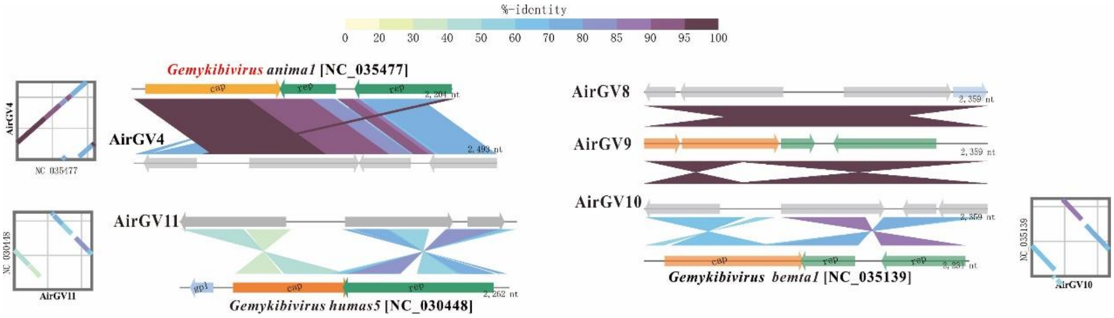

Figure S9 Genomic alignments of AirGVs 4 and 8-11 with their closest references. Pairwise dot plots of these genomes are shown. Pairwise dot plots of these genomes are shown. AirGVs 4 and 8–11 share 50–100% identity with partial sequences of known *Gemykibivirius* genomes.

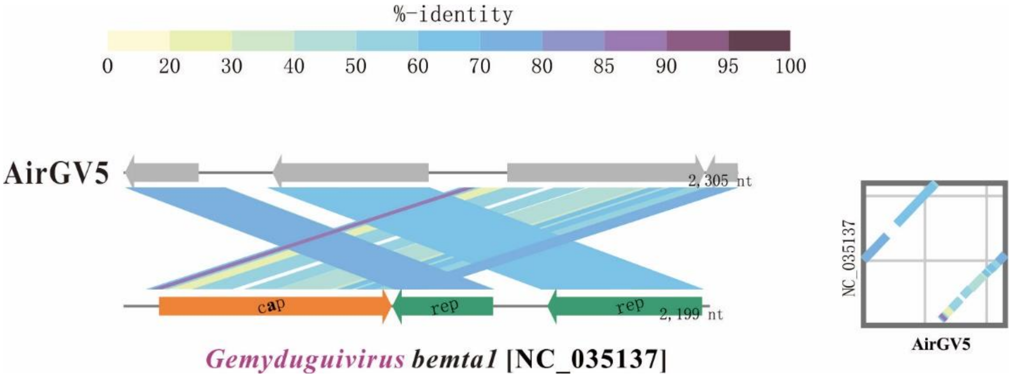

Figure S10 Genomic alignments of AirGV5 with the closest reference *Gemyduguivirus bemta1* phage (NC_035137). Pairwise dot plots (right) of these genomes are shown. AirGV5 shares 50–60% identity with partial sequences of known *Gemyduguivirus bemta1* genome.

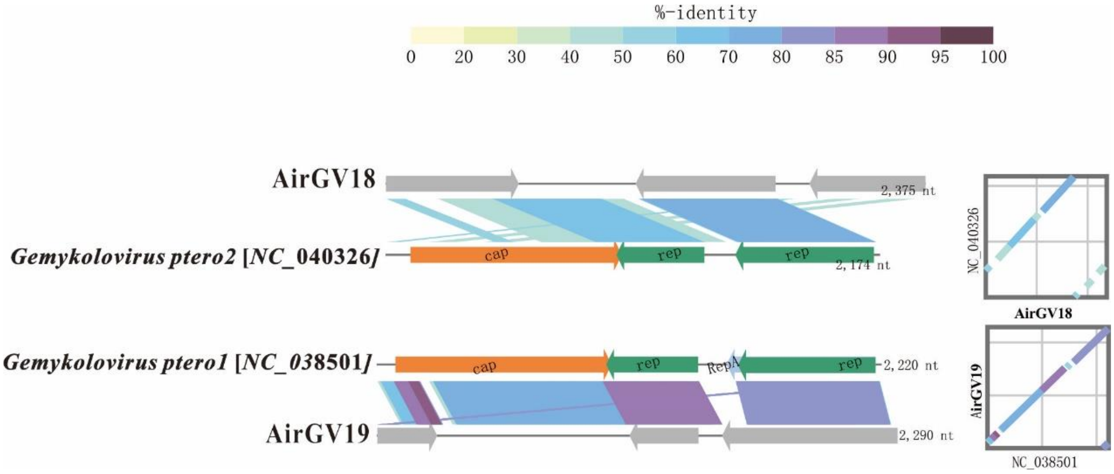

Figure S11 Genomic alignments of AirGV18 and AirGV19 with the closest references *Gemykolovirus ptero* phage (NC_040326 and NC_038501). Pairwise dot plots (right) of these genomes are shown. AirGV18 and 19 share 50–90% identity with partial sequences of known *Gemykolovirus* genomes.

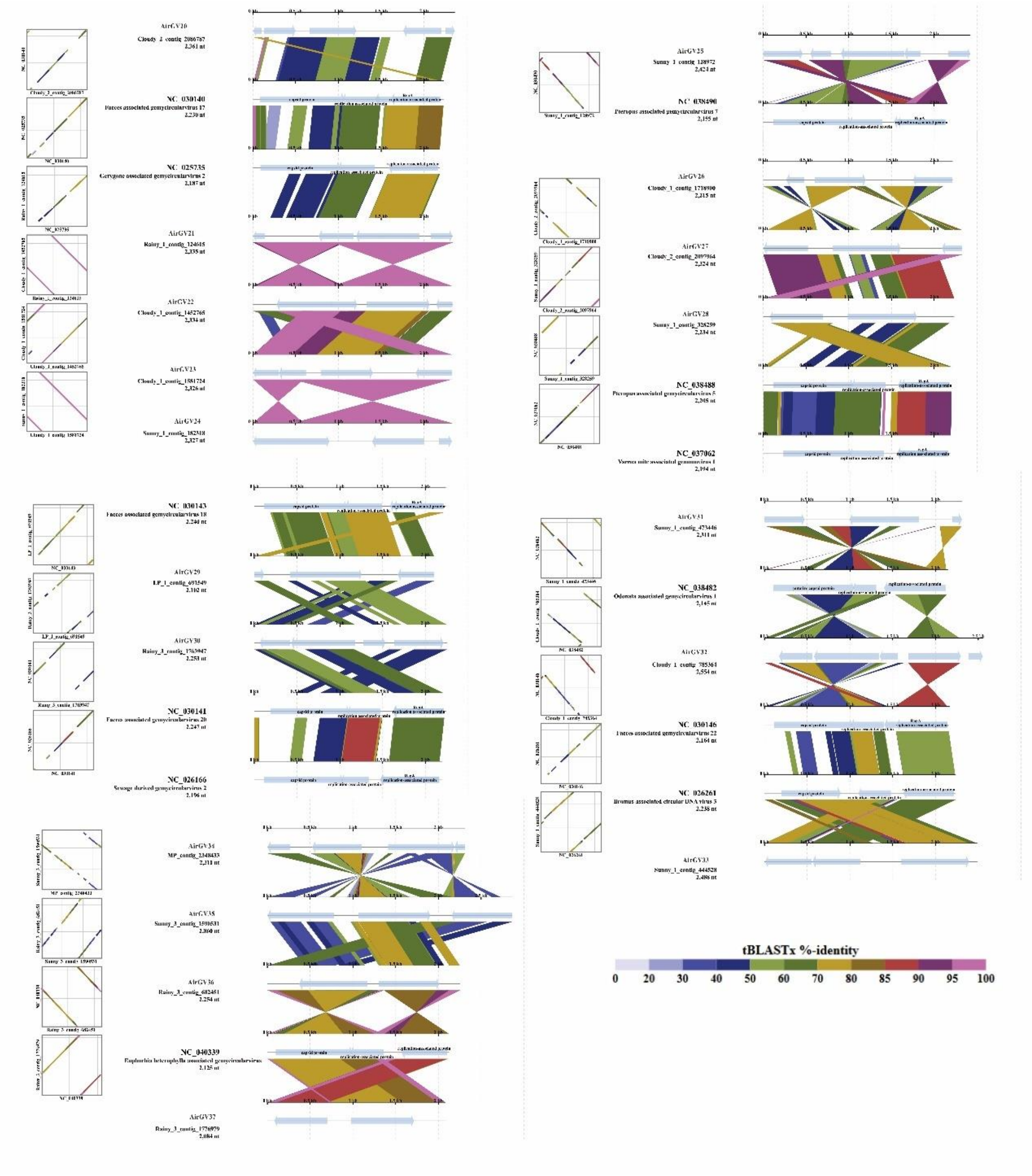

Figure S12 Genomic alignments of AirGVs 20-37 with their closest references *Gemycircularvirus* phages. Pairwise dot plots of these genomes are shown. AirGVs 20-37 share 50–100% identity with partial sequences of known *Gemycircularvirus* genomes.

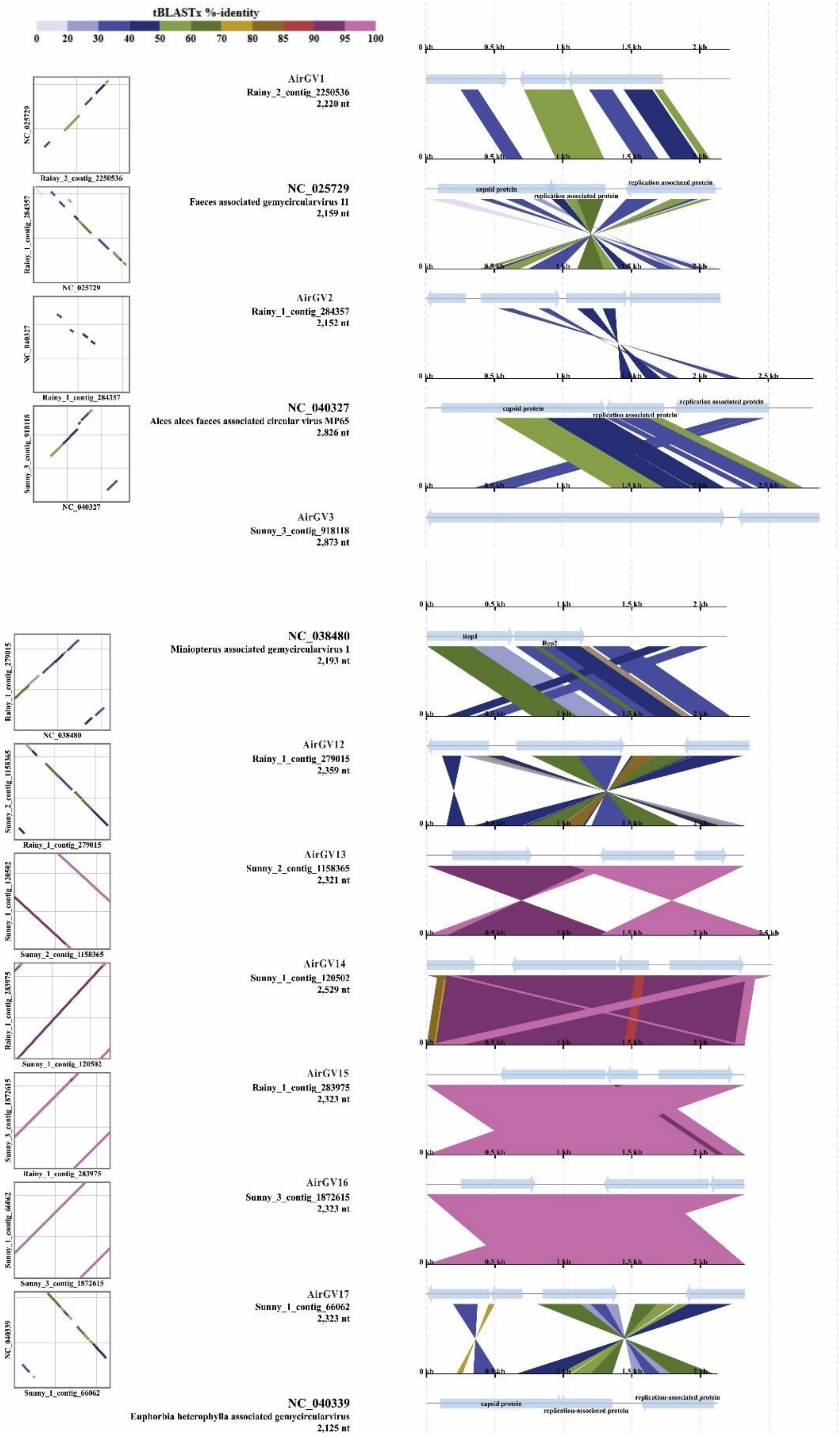

Figure S13 Genomic alignments of AirGVs 1-3, 12-17 and their closest genomoviruses. Pairwise dot plots of these genomes are shown. The complete genomes of the 9 AirGVs showed 30–60% similarity with known viruses.

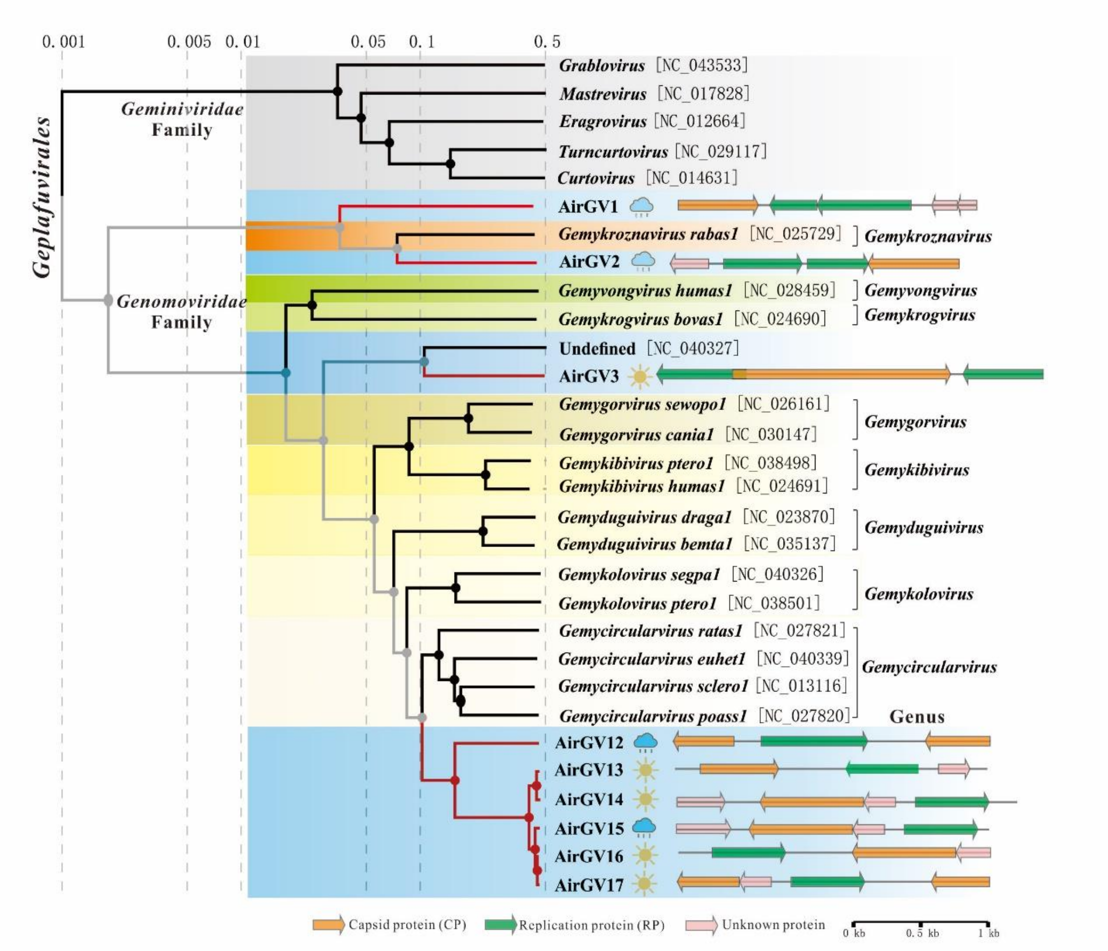

Figure S14 Phylogenetic tree of 9 new AirGVs with low identities with available genomoviruses. Genomic organizations of AirGVs are shown in the tree. Sequence similarity among the representative genomoviruses and AirGVs are presented in Figure S13. AirGVs 12–17 from rainy and sunny days clustered into a new group at the genus level, for their 39–55% similarity with known viral genomes and 42–46% similarity to known Rep proteins.

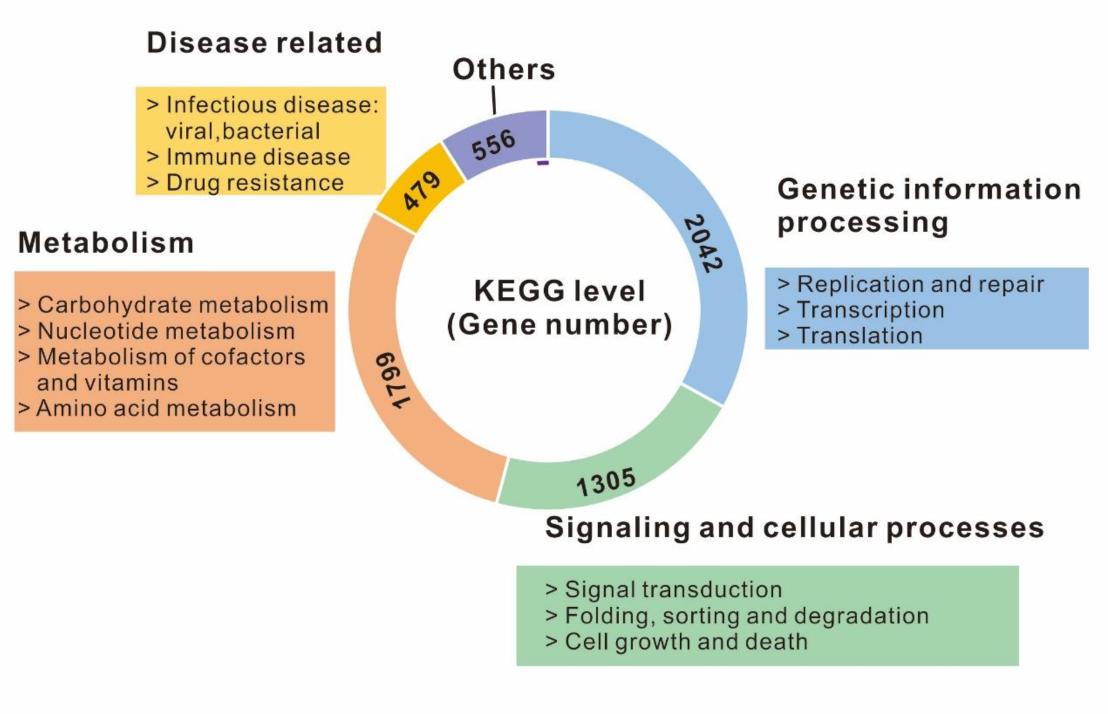

Figure S15 Predicted functional genes of the airborne viromes with confident contigs (1.5–184.2 kb). The KEGG database (https://www.kegg.jp/) was used to analyze gene functions (e ≤ 1e–3) and biological systems, such as genetic information processing, signaling/cellular processes, metabolism and functions related to infectious/immune diseases.

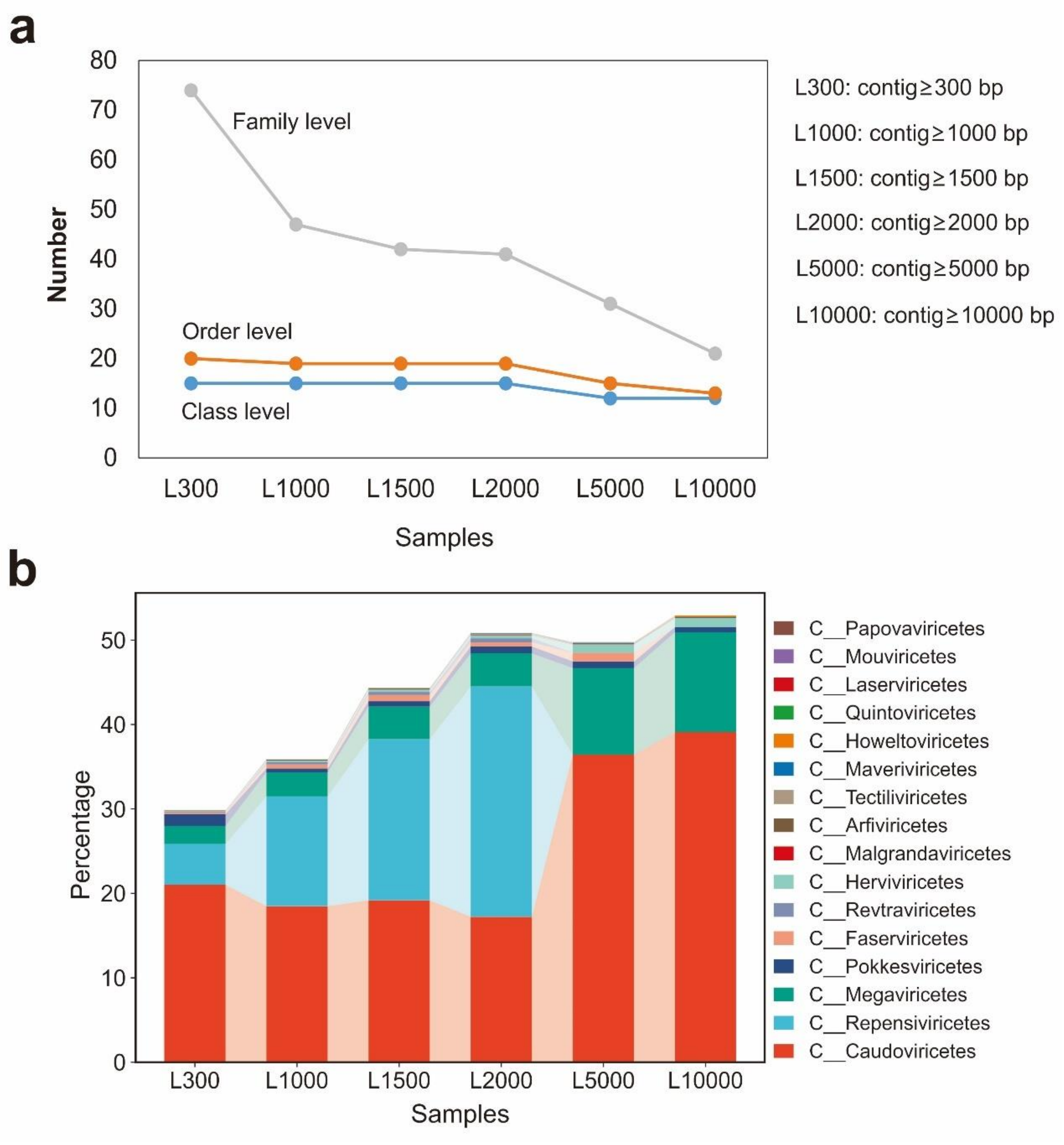

Figure S16 The diversity and abundance of viral community with different sequence sizes of contigs. **a.** The diversity of viral community at class/order/family levels. Viral metagenome diversity was decreased as contig length increased, especially at the family level. The diversity of viral communities with contigs ≥ 1.0, 1.5 and 2.0 kb had no significant difference at class and order levels. Contigs ≥ 5.0 and 10.0 kb had a significant decrease in viral diversities at various taxonomic levels. **b.** The abundance of viral community at the class level. Although the diversity of viral communities showed minor differences at the class and order levels, the viral abundance and

structure varied with contig length. Among the high-confidence viral contigs with classified and unclassified viruses, the three most abundant viruses varied in different ways. The abundance of viruses assigned to dsDNA *Megaviricetes* increased with contig length. The abundance of dsDNA *Caudoviricetes* decreased from 0.3- to 2.0-kb contigs, whereas their abundances greatly increased with sequences larger than 5.0 kb. The trend of change in ssDNA *Repensiviricetes* was completely opposite to that of *Caudoviricetes* from 0.3- to 2.0-kb contigs. *Repensiviricetes* did not exist in these viral communities with sequences larger than 5.0 and 10.0 kb.
